# Modeling and Mechanistic Study of Polyethylene Chain Cleavage during Ball Milling

**DOI:** 10.1021/acs.macromol.5c02110

**Published:** 2025-10-10

**Authors:** Tobias Morgen, Stefan Mecking, Ina Vollmer

**Affiliations:** † 26567 University of Konstanz, Chair of Chemical Materials Science, Department of Chemistry, Konstanz 78464, Germany; ‡ 84889 Utrecht University, Inorganic Chemistry and Catalysis, Institute for Sustainable and Circular Chemistry, Department of Chemistry, Utrecht 3584CG, The Netherlands

## Abstract

Mechanochemical conversion of polyethylene (PE) and polypropylene was shown to produce monomers and is thus interesting for chemical polymer recycling. As these polymers make up more than 50% of the worldwide polymer production, studying their conversion during ball milling is especially relevant. However, fundamental knowledge on the effect of crystallinity, degree of polymerization, entanglement and temperature on the conversion is lacking due to the difficulty in producing polyolefins with controlled chain length and dispersity. Here we synthesize PE by a controlled chain growth polymerization and study its degradation during ball milling at cryogenic conditions, at room temperature (RT), with and without air, and using either steel or zirconia grinding spheres. Resulting molecular weight distributions are fitted using a statistical chain cleavage model suggesting a statistical Gaussian distribution of chain cleavage probability around the middle of the chain. This is likely because if the chains are fixed in an entanglement or crystal at two points, they cannot slip out and force can act on them leading to cleavage. That the chain is fixed at both sides of a possible cleavage location is most likely if the cleavage location is in the center of the chain. Chain cleavage is also promoted when entangled domains exist that link crystalline regions. A micelle grown single crystal ultrahigh molecular weight PE without entanglements was milled and its molar mass decreased much less compared to the same sample that was annealed to create entanglements. However, in contrast to previous studies and common expectations, the initial molar mass of the polymer, the degree of crystallinity and the brittleness of the sample did not have a measurable influence on chain cleavage. While the decrease in molar mass was faster at cryogenic conditions compared to RT, nuclear magnetic resonance (NMR) results suggest that this is due to a suppression of radical recombination rather than the higher brittleness of the material below its glass transition temperature (∼−120 °C). Similarly, the number of permanent scissions increased by up to 2.6 times under air compared to nitrogen atmosphere, especially in combination with steel milling spheres. NMR spectra of the milled samples suggest that the reaction of mechanochemically formed chains with air suppresses recombination.

## Graphical abstract

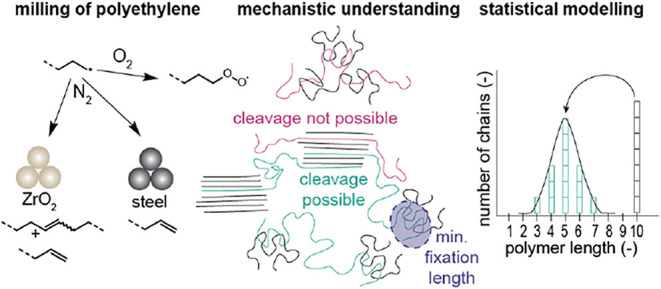

## Introduction

While initially being studied out of interest to prevent degradation,^1,2^ mechanochemical degradation of commodity polymers is gaining attention as a potential method for recycling.^3,4^ It was shown that even ball milling of polymers with a sole C–C backbone, like poly­(methyl methacrylate) (PMMA), polystyrene (PS),^5,6^ polypropylene (PP),^7^ and polyethylene (PE)^7^ can produce monomers. The collision of grinding spheres upon agitation in the ball mill impacts polymer trapped at the collision point leading to homolytic cleavage of the polymer chains and the formation of two radicals. Radical formation was observed by electron spin resonance spectroscopy even during milling in liquid N_2_.^8,9^ These radicals can undergo termination via recombination or disproportionation, of which only the latter leads to permanent scission and a decrease in molar mass.^10^ Kinetic parameters derived from thermodynamic information and rate constants of model compounds suggests that recombination is 10 times faster than disproportionation.^11^ In view of formation of monomer from the primary radicals, the desired reaction is β scission. However, for example for PP, only every 30th radical formed was shown to release a monomer.^12^

The initial chain cleavage rate is influenced by the polymer morphology and the milling conditions. For fully amorphous polymers like PS and PMMA, the glass transition temperature (*T*_g_) was shown to influence the cleavage rate.^13^ The *T*_g_ of these polymers lies above room temperature (RT). A correlation was found where a higher *T*_g_ leads to faster cleavage at RT. Cleavage seems to be facilitated in the glassy state as impact energy cannot easily be dissipated. In the rubbery state, polymers can disentangle upon impact instead of undergoing chain cleavage. However, the *T*_g_ of PP and PE is far below RT. Therefore, one would expect a higher cleavage rate at cryogenic conditions. In addition, PE and PP are partially crystalline. The question arises as to whether the very rigid crystalline or the amorphous regions would be more susceptible to impact induced chain cleavage. The structure of these semicrystalline polymers can be much more complicated (Scheme 1) than that of fully amorphous materials. Usually, polymer crystals are not separated from each other and are not perfect. Several individual chains are part of one crystal while also protruding into amorphous regions. Polymer chains can span several crystals as tie molecules and crystals can be connected by entanglements. However, it was shown that when synthesized in very diluted media, single PE chains are separated and therefore form single chain disentangled nanocrystals.^14^ For example, in an aqueous emulsion polymerization, single crystals of uniform shape were synthesized with a long-lived Ni­(II) catalyst.^14^ In this case, the crystal size depends on the molar mass, which can be tuned by selecting a suitable polymerization time.

**Scheme 1:**
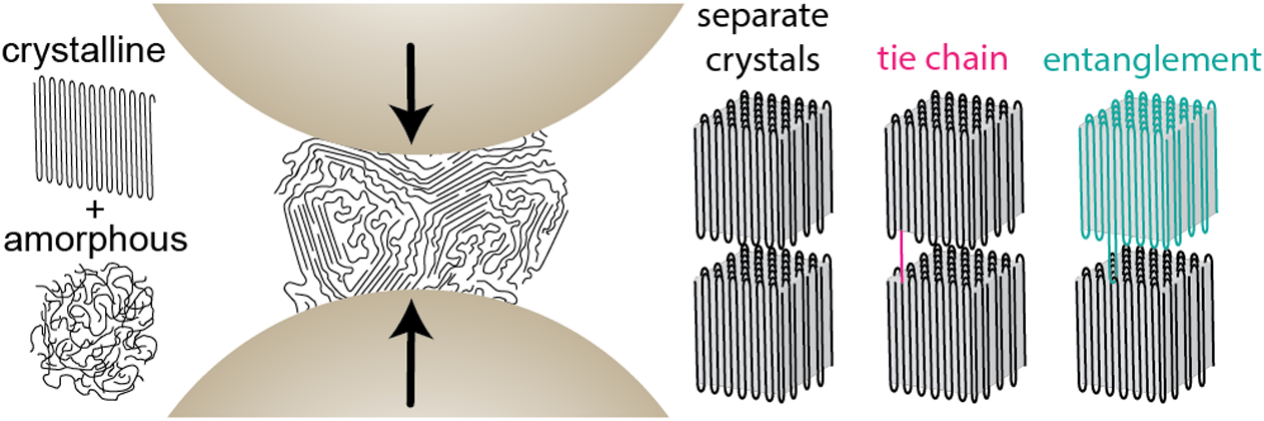
Amorphous and Crystalline Regions in A Polymer Sample and the Connection of Crystals by Tie Chains

The effect of the degree of crystallinity on the cleavage rate was studied by varying the tacticity of polylactic acid (PLA). It was found that the polymer is amorphized within minutes of milling and therefore the initial degree of crystallinity did not impact the chain cleavage rate.^15^ However, for other polymers, it was found that the mechanochemical degradation rate also depends on the initial molar mass of the polymer^16^ and that no further bond scission is observed for polymers below a polymerization degree of around 100.^6^ It was proposed that this is because van der Waals interactions between protons are needed to prevent the polymer chains from slipping past each other. For PE, it was calculated that at a degree of polymerization of 83, these interactions add up to a value close to the backbone C–C bond strength.^1^ Experimentally, a value between 71 and 100 was observed for mechanochemical degradation of PE in an extruder.

Simulations can be used to study the cleavage location along the length of the polymer chains. The results are often compared to degradation kinetics or molar mass distributions. The chain cleavage probability can be represented with different models: (a) random distribution of chain cleavage locations along the chain of the polymer, (b) detachment of monomers from the chain end (c) or a chain cleavage probability of Gaussian form centered around the middle of the chain.^15^ It was shown that (c) is the best model for PS and PMMA cleaved during ultrasonication and ball milling by comparing simulation results with molar mass distributions obtained from size exclusion chromatography (SEC) after degradation of the polymers.^13,17^ Other authors have also simulated thermal cleavage^18^ and cleavage under shear in an extruder.^19^ However, to the best of our knowledge, none of the studies have directly fitted experimentally obtained molar mass distributions by adjusting the parameters of such models.

Whether chain cleavage during ball milling also occurs in the middle of the chain for PP and PE is not yet known. As PE and PP are partially crystalline in contrast to the fully amorphous PS and PMMA, studied previously, the morphology could greatly affect the cleavage location. For example, a tie molecule could be broken on the edge of a crystalline region rather than in the middle of the chain. Studying the chain cleavage of these polymers is difficult as commercial polymers usually have a rather wide molar mass distribution (*M*_w_/*M*_n_ > 3), which smears out shifts in molecular weight.^7^ Modeling of the chain cleavage position is only reliable with polymers that have a low *M*_w_/*M*_n_. PMMA and PS with narrow molecular weight distributions can easily be obtained by various controlled ionic and radical polymerization techniques. In contrast, PE and PP are mostly produced by Ziegler–Natta and Phillips, as well as, more recently metallocene catalysts with which *M*_w_/*M*_n_ ∼ 1 cannot be achieved.^20^ However, there are some nonmetallocene Ti based homogeneous catalysts allowing for unprecedented control and high molar masses in ethylene solution polymerizations.^21^ Weakly coordinating aryl *o*-F substituents of Ti prevent contact with the β-CH_2_ of the growing polymer chain, thereby suppressing β-hydrogen elimination that usually leads to broad molar mass distributions.^22,23^

Here we adapt a chain cleavage model previously used to simulate the mechanochemical degradation of PS^17^ and star polymer^24^ solutions during ultrasonication to simultaneously fit molar mass distributions obtained after various time points of ball milling. We perform the fitting for milling under air and N_2_, at RT and under cryogenic conditions, as well as, with steel and zirconia grinding spheres. To enable meaningful fits, we synthesize PE of various molar mass, all with a low *M*_w_/*M*_n_. In addition, we study the effect of entanglements in ultrahigh molecular weight PE (UHMWPE) on the chain cleavage efficiency during milling by comparing a disentangled UHMWPE^14^ and an annealed material that contains entanglements.

## Experimental Section

### PE Synthesis

PE synthesis was carried out in a 500 mL glass reactor using a magnetic stirrer. The reactor was kept at RT by immersing it in a water bath. The reactor was purged with nitrogen three times with subsequent evacuation, while heating it with a heat gun. Subsequently, distilled toluene (300 mL) was introduced and stirred vigorously with a Teflon stir bar. Continuous and intensive stirring during the reaction is crucial to obtain a PE with a narrow molecular weight distribution. The reactor was quickly evacuated and then the ethylene supply was opened. The ethylene pressure was constantly kept at 1 atm during the whole polymerization. After the solution was saturated with ethylene for 15 min, the polymerization was initiated by fast addition of a toluene solution (ca. 10 mL) of modified methylaluminoxane (MAO) and the Ti complex using a syringe, previously prepared in the glovebox. After the desired reaction time, the ethylene gas feed was stopped and 50 mL HCl (37 mol %) were added to terminate the polymerization reaction. The reaction mixture was left stirring for 60 min and then added to acidified methanol (25 mL of concentrated HCl in 500 mL of methanol). Solid PE was recovered by filtration, thoroughly washed with methanol, and dried at 50 °C for 24 h in a vacuum oven. Exact synthesis conditions for each PE sample can be found in Table S1.

### Disentangled PE

Disentangled PE (PE_single_) was synthesized according to Schnitte et al.^14^ with a reaction time of 5 h using 7.5 μmol of C_6_F_13_-tetrasubstituted N-terphenyl salicylaldiminato Ni polyethylene glycol (PEG, 5 kg/mol) catalyst and 12 g of sodium dodecyl sulfate (SDS), 3 g of CsOH, 1.5 mL toluene as additive, a temperature of 10 °C and a pressure of 40 bar yielding 23.95 g of polymer.

### Annealing

Annealing was performed in a septum sealed vial flushed with nitrogen through a needle, containing 300 mg of PE_single_. The vial was placed in a heating block kept at 135 °C for 3 h yielding PE_anneal_.

### Ball Milling Experiments

Ball milling experiments were performed in a Retsch CryoMill with a 25 mL steel jar (Retsch), using five grinding spheres of 10 mm diameter, either made of steel (Retsch) or zirconia (Zhonglong Materials). A milling frequency of 30 Hz was used for all experiments. Experiments performed for longer than 1 h were performed in 1 h intervals with 30 s pauses. For milling under N_2_, the milling jar was filled in the glovebox and closed tightly, while for milling under air, the jar was just filled in the open. Milling at RT denotes milling without cooling. Due to friction during milling, the milling jar temperature increases up to 45 °C. Milling at cryogenic temperatures was performed with the automatic liquid N_2_ dosing system of the device keeping the jar constantly at −196 °C. The samples were first autoprecooled to the desired low temperature while shaking with only 5 Hz and then milled at 30 Hz as already described. For radical trapping experiments, either 30 mg di-*tert*-butyl hydroxytoluene (BHT) or 21 mg 2,2,6,6-tetramethylpiperidinyloxyl (TEMPO) were added to the grinding jar together with the polymer.

### Differential Scanning Calorimetry (DSC)

Differential Scanning Calorimetry was performed on a Netzsch Phoenix 204 F1 at a heating/cooling rate of either 1 or 10 °C/min with 4–6 mg of polymer in an aluminum pan sealed in the open air. Experiments with 1 °C/min were performed in one heating phase from RT to 180 °C, whereas measurements with 10 °C/min comprised heating to 180 °C, cooling to −50 °C followed by a second heating phase to 180 °C. Polymer crystallinities were calculated from the heating curves based on a melt enthalpy of 293 J/g for 100% crystalline PE.

### High Temperature Size Exclusion Chromatography (HT-SEC)

High Temperature Size Exclusion Chromatography was performed on a PolymerChar GPC-IR instrument equipped with PSS Polefin Linear XL columns (3 × 30 cm), an additional guard column, a four-capillary viscometer and an IR5 dual wavelength detector (selective for methylene and methyl units) with 1,2-dichlorobenzene as solvent with a flow rate of 1 mL/min at 160 °C. All samples were dissolved by shaking in hot 1,2-dichlorobenzene at 160 °C for 90 min prior to injection. For samples with *M*_n_ < 200 kg/mol the concentration of the injected solution was 0.5–2.0 mg/mL and a linear calibration with monodisperse PE standards was employed. UHMWPE and its respective milled samples were measured with only 0.5 mL/min and evaluated by linear calibration with monodisperse polystyrene standards. The concentration for these samples was 0.3–0.5 mg/mL.

### High Temperature Nuclear Magnetic Resonance Spectroscopy (HT-NMR)

High Temperature Nuclear Magnetic Resonance Spectroscopy was performed on a Bruker Avance III HD 400 spectrometer (^1^H: 400 MHz) in 1,1,2,2-tetrachloroethane-*d*_*2*_ at 100 °C. The NMR solvent was deoxygenated by bubbling with N_2_ for 30 min. All NMR samples were prepared and sealed in a glovebox to ensure that samples do not oxidize during the long high temperature NMR measurements. The proton chemical shifts were referenced to the residual solvent proton signals (C_2_Cl_4_D_2_: 6.00 ppm). Chemical group densities are given in mol % with respect to the total number of CH_2_ groups of the polymer backbone.

## Results and Discussion

We synthesized various PEs of different number average molar mass (*M*_n_), all narrowly distributed (Table 1) and milled them under air and N_2_, at RT and under cryogenic conditions, as well as, with steel or zirconia grinding spheres.

**Table 1:** Polymer Notation Together with *M*_n_, *M*_w_ and Their Ratio Determined by HT-SEC, as Well as, Melting Points and Degrees of Crystallinity Determined by DSC

	*M*_n_	*M*_w_	*M*_w_/*M*_n_	melting point [°C]		crystallinity [%]	
polymer notation	[g/mol]	[g/mol]	[-]	1st heat	2nd heat	1st heat	2nd heat
PE_103_	102,900	119,600	1.16	143.6	139.0	74.6	59.9
PE_46_	45,700	49,800	1.09	143.5	137.3	81.3	70.6
PE_56_	56,000	61,300	1.10	144.4	139.0	82.2	66.9
PE_160_	159,400	199,500	1.25	144.3	138.2	80.6	60.3
PE_170_	169,800	186,400	1.10	143.6	139.7	79.0	57.6
PE_single_	1,566,100	3,059,200	1.95	145.3	136.7	71.7	41.0
PE_anneal_	n.d.	n.d.	n.d.	133.5	135.0	53.4	58.8

Upon milling, the polymer chains are cleaved, and the molar mass distributions continuously shift to lower molar masses (Figure 1a). In the first 0.5 h, a second peak appears at lower mass and over time only this second peak remains. This causes the *M*_n_ (Figure 1b) and weight average molar mass (*M*_w_) to decrease over time, while the ratio *M*_w_/*M*_n_ increases (Figure 1c). The limiting molar mass of 2.8 kg/mol, corresponding to a limiting degree of polymerization of 100, is almost reached for the harshest milling conditions of cryogenic milling under air with steel grinding spheres. Milling at RT denotes milling without cooling. Due to friction during milling, the milling jar temperature increases up to 45 °C.

**Figure 1:**
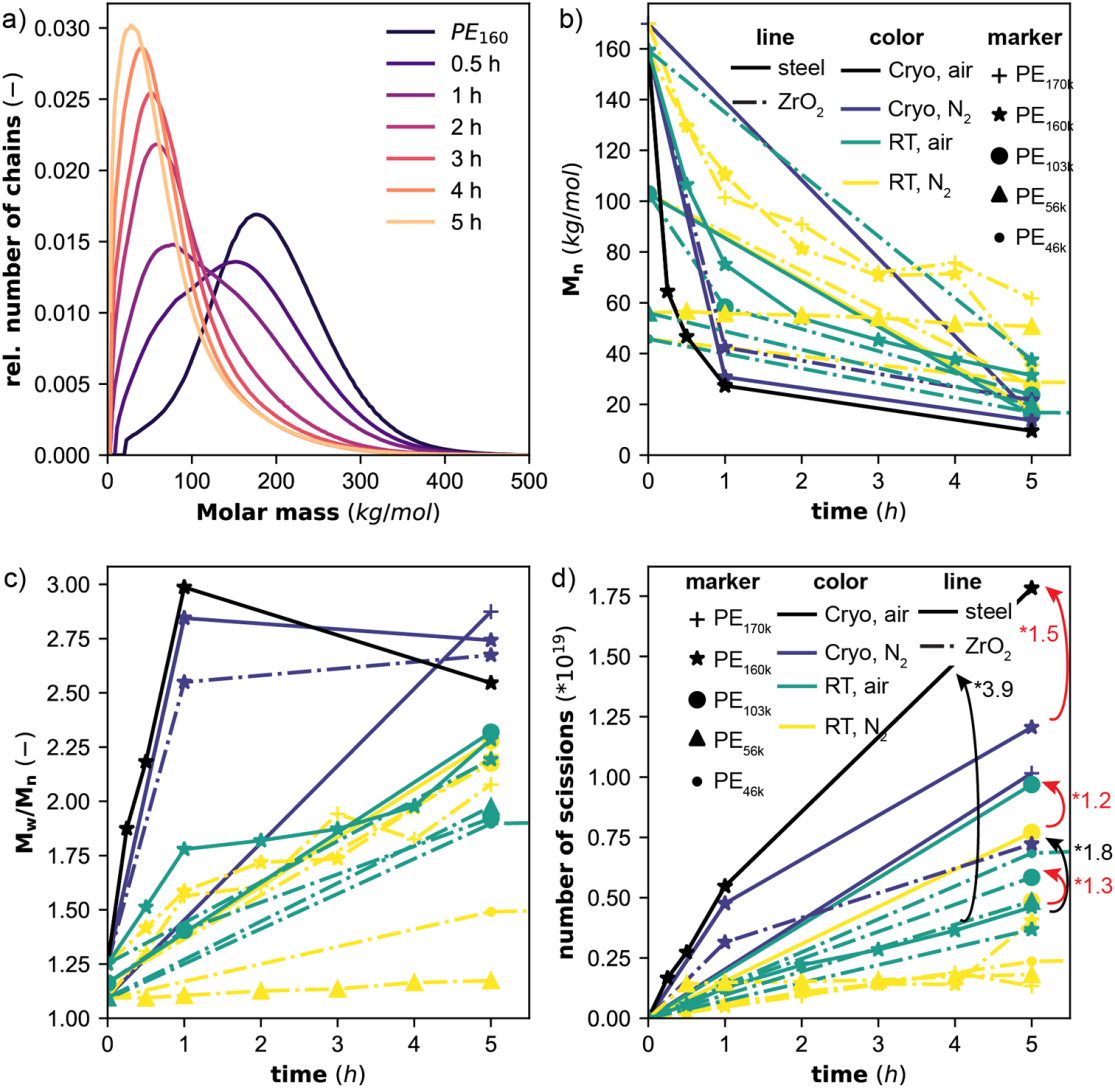
(a) Molar mass distributions obtained from HT-SEC before and after milling of 300 mg of PE_160_ for 0.5, 1, 2, 3, and 5 h at 30 Hz under air at RT in a 25 mL steel container, using 5 ZrO_2_ grinding spheres (10 mm). (b) *M*_n_, (c) *M*_w_/*M*_n_, and (d) number of permanent scissions, before and after milling under various conditions. The red arrows indicate the increase in permanent scissions with air compared to N_2_ atmosphere, while the black arrows indicate an increase in permanent scissions at cryogenic compared to RT conditions. Milling at RT denotes milling without cooling. Due to friction during milling, the milling jar temperature increases up to 45 °C.

Knowing the total weight of polymer used (*m* = 300 mg), the total number of polymer chains in the system and the number of cleavages is calculated (eq 1, Figure 1d), where *N*_A_ is the Avogadro constant and *N*_0_ is the number of chains present in the original polymer sample. The decrease in *M*_n_ is faster at shorter milling times and subsequently slows down.

1 $$N_{t}=\frac{m}{M_{n}}\cdot N_{A}-N_{0}$$

Several polymers with different *M*_n_ values were milled at the same conditions (RT, N_2_, ZrO_2_ spheres). All have the same degradation rate, meaning that the initial *M*_n_ does not influence the degradation rate. This is in contrast to what was observed during ultrasonication,^25^ but in line with observations during ball milling of PS and PMMA, in which the glass transition temperature of the polymer determined the degradation rate, rather than *M*_n_.

The terminal double bonds expected to form upon disproportionation are observed with ^1^H NMR (Figure 2a,c). However, internal double bonds are also observed, which are likely to result from inter- or intramolecular H-transfer prior to disproportionation (Scheme 2).

**Figure 2:**
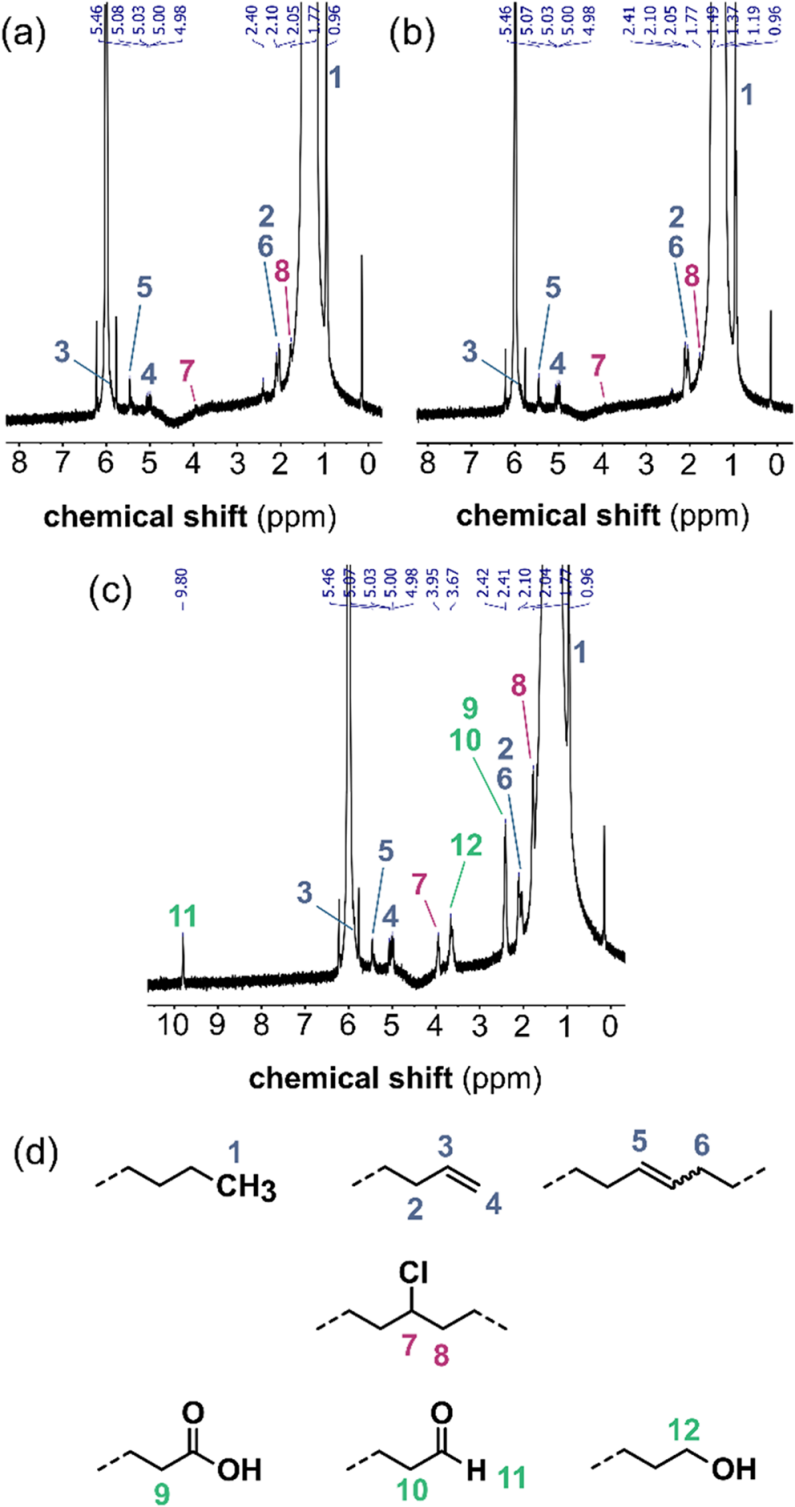
^1^H NMR spectra (400 MHz, 100 °C, C_2_D_2_Cl_4_) of milled PE_160_ (a) 5 h, cryogenic, N_2_, 5 zirconia grinding spheres (b) 5 h, cryogenic, N_2_, 5 steel grinding spheres and (c) 5 h, cryogenic, air, 5 steel grinding spheres. (d) Signal assignment to different functional groups. The chlorinated PE backbone stems from reactions of the polymer with the NMR solvent during the long high-temperature measurements required for detection of end groups.

**Scheme 2:**
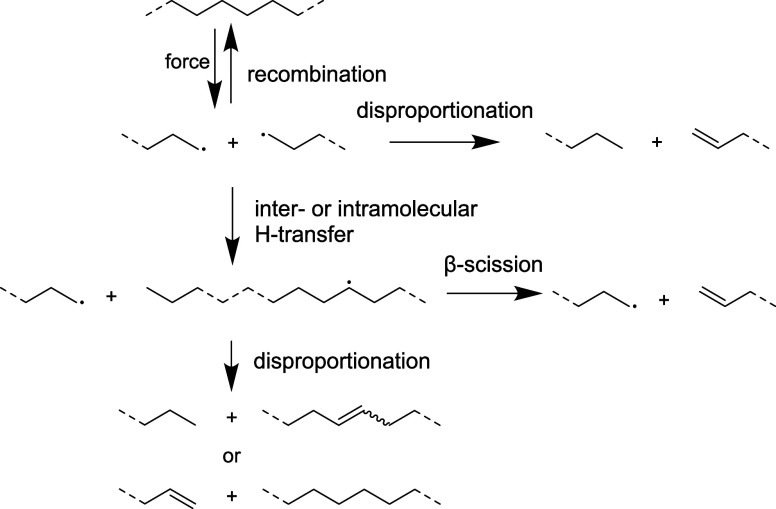
Suggested Propagation Mechanism Proceeding after Mechanochemical Chain Cleavage, Leading to an Overall Decrease in Molar Mass, the Creation of New Chains and Internal as Well as Terminal Double Bonds

### Effect of Temperature on Degradation

More new chains are formed under cryogenic conditions than during milling at RT. For example, with PE_160_ under N_2_ with ZrO_2_ grinding spheres 1.8 times more chains are formed, while under air with steel grinding spheres 3.9 times more chains are formed (black arrows, Figure 1d). At cryogenic temperature, the amorphous regions of the PE are glassy. This could mean that the impact energy dissipates less easily through the polymer, therefore leading to higher stress concentration causing more cracks and thus chain cleavage. However, this implies that the chain cleavage probability along the chain should be different at RT and under cryogenic conditions. This is not the case as is discussed below.

Another effect could be that the lower mobility of the chains separates radicals spatially and, thus, prevents recombination reactions (Scheme 2). If recombination was suppressed, a faster chain cleavage rate would be expected. Radical trapping with BHT leads to the formation of 1.17 more chains, while trapping with TEMPO leads to 3.19 times more chains after 5 h of milling PE_170_ at cryogenic conditions using steel spheres (Figure S1). The more pronounced effect when adding TEMPO compared to BHT could be due to the lower trapping efficiency of BHT or the initiation of more chain cleavage by hydrogen abstraction from PE by TEMPO. Also, kinetically, recombination is suppressed at lower temperature. That is, its reaction rate constant is about 4 orders of magnitude lower at cryogenic conditions (Table S2).^11^ This allows the chains even more time to separate from each other.

### Faster Decrease in Molar Mass under Air

Milling under air leads to a 1.2 (PE_103_, steel, RT) to 2.7 (PE_160_, steel, Cryo) times faster formation of new chains. The accelerated formation of new chains under air could be explained by a stabilization of radical chain ends and suppression of recombination due to reaction with O_2_ similar to what was previously observed with milling of PS.^6^ Peroxy-radicals could form upon reaction of a polymer radical with O_2_ and follow-up reactions could lead to other oxygen containing groups (Scheme 3). Indeed, alcohol, aldehyde and carboxylic end groups are found by ^1^H NMR in the milled residue (Figure 2c) that are not present in the unmilled polymer (Figure S2). Peroxy-groups are not observed, likely because they are quite reactive and undergo further reactions already under milling conditions. It should be noted that samples milled under air also contain internal and terminal double bonds, meaning that the mechanism of Scheme 2 is also operant to some extent when O_2_ is present.

**Scheme 3:**
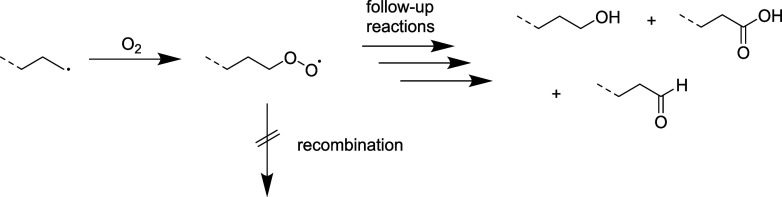
Suggested Mechanism of Polymer Chain End Radicals Reacting with Oxygen

If every chain reacts with O_2_, the expected concentration of oxygen containing end groups referenced to CH_2_ would be 0.28 mol % which is indeed very low and approximately matches the obtained results (0.22 mol % for the sample in Figure 2c). The oxygen availability in the container could limit this effect. However, enough oxygen seems to be present. Prior to milling, 21.5·10^–5^ mol of O_2_ are available in the 25 mL container, while after 5 h of milling with steel spheres at cryogenic conditions under air, 2.96·10^–5^ mol chains are produced. Note that, as expected, for both samples milled under oxidative and inert atmosphere, the total number of chain ends vs methylene groups shows a linear correlation with the respective *M*_n_ determined by HT-SEC (Figure S6).

### Promoting Effect of Steel Spheres

Using steel compared to zirconia spheres leads to 1.7 times more chains under air and 1.6 times more chains under nitrogen with PE_103_ after 5 h of milling at RT. Also, for 5 h of cryogenic milling of PE_160_ under N_2_, a 1.7-fold increase was observed. ^1^H NMR shows a similar 1.8-fold increase of the double bond concentration (Figure 2a,b).

The fact that a similar stabilizing effect was observed under both oxidative and inert atmosphere suggests that polymer radicals might interact directly with the surface of the steel spheres and that an interaction with O_2_ is not required in contrast to what was suggested previously for milling PS with steel spheres under O_2_.^5^ This is further supported by ^1^H NMR (Figures 2a,b and S5), which shows that also under N_2_ the number of terminal double bonds increases from 0.008 to 0.021 mol % after 5 h of cryogenic milling with steel compared to zirconia spheres. This suggests that inter- and intramolecular hydrogen transfer (Scheme 2) is suppressed when milling with steel spheres, which could be a result of stabilization of chain end radicals on the steel surface. A similar stabilization effect was proposed for milling with surface activated zirconia grinding containing oxygen defects and Zr^3+^ species.^7^

### Cleavage Probability Is Distributed around the Center of the Chain

To determine the location along the polymer chain, where cleavage probability is highest, we adapted a model from Glynn et al.^17^ The model predicts the molar mass distribution after a certain number of cleavage steps based on the assumption that longer chains are cleaved with a higher probability and that cleavage probability is distributed around the center of the chain. The model was implemented in python and can be found in the accompanying jupyter notebook.^26^ An extended explanation of the procedure can be found in the SI.

In the first step of the model algorithm, certain chains are selected from the distribution, represented as a probability distribution curve (Figure S7a). This curve is calculated assuming that the probability of cleavage increases with molar mass (eq 2), which is the degree of polymerization *x* multiplied by the monomer molar mass $M_{0}=28\frac{g}{\mathrm{mol}}$ . The dependence is stronger for larger values of the exponent *s*. Equation 2 is multiplied with the original molar mass distribution *f*_n_ to obtain the distribution curve of chains to select for cleavage (eq 3). At the first simulation step, the molar mass distribution of the original polymer, obtained from HT-SEC, is used as input.

2 $$C={\left(M_{0}\cdot x\right)}^{s}$$

3 $${P\left(t,x\right)=f}_{n}\cdot C$$

The script iterates over the polymerization degree *x* and calculates *P*(*t*,*x*). It also calculates how many chains of polymerization degree *x* are produced by cleavage of chains larger than *x*, starting from *y* = *x* + 1 (eq 4).

4 $$\sum_{y=x+1}^{\infty }P\left(t,y\right)Q\left(y,x\right)$$

5 $$Q\left(y,x\right)=\frac{1}{\mathrm{ry}\surd 2\pi }e^{\left(-{\left(x-\frac{y}{2}\right)}^{2}/2r^{2}y^{2}\right)}$$

*P*(*t*,*y*) is calculated the same way as *P*(*t*,*x*) (eq 3) and *Q*(*y*,*x*) is a Gaussian distribution centered around the polymerization degree *y*/2 (eq 5). Here, *ry* represents the width of the Gaussian distribution, equivalent to what is usually denoted as the standard deviation σ. A larger *r* represents a cleavage that is more broadly distributed around the center. However, this width of the distribution also increases with the degree of polymerization *y* (Figure S7b).

At each simulation step *t*, the process is repeated by iteration over all *x* and a new chain length distribution is obtained via eq 6. Refer to Glynn et al. for the derivation.^17^

6 $$f_{n}\left(t+1,x\right)=\frac{\left(N+t\right)f_{n}\left(t,x\right)-P\left(t,x\right)+2\left\{\sum_{y=x+1}^{\infty }P\left(t,y\right)Q\left(y,x\right)\right\}}{N+t+1}$$

Here *N* represents the number of chains, where in each simulation step a certain fraction of chains is cleaved, roughly scaling with 1/(*N* + *t*). In our adaptation of the model, *r* in eq 5 is varied together with *s* in eq 2 to fit the measured molar mass distributions after certain milling times.

The fact that the model can fit the molar mass distributions after different milling conditions satisfactorily (Figures 3 and S10–S27) is a strong indication that the chains are cleaved most likely in the middle of the chain. An even better fit is obtained for the development of *M*_n_ and the number of scissions over time. The width of the Gaussian (*r* = 0.10–0.18, Table S3) is similar to what has been observed in ultrasonication of polymer solutions (*r* = 0.15).^17^ Smaller values of *r* are obtained for very high degrees of degradation after 5 h of milling under cryogenic conditions. However, in this case the fits are not reliable (Figures S12, 13, 15, and 24). And when only fitting the first hour of milling under cryogenic conditions, *r* values in a similar range as for RT milling are obtained (entries 5 and 7 in Table S3).

**Figure 3:**
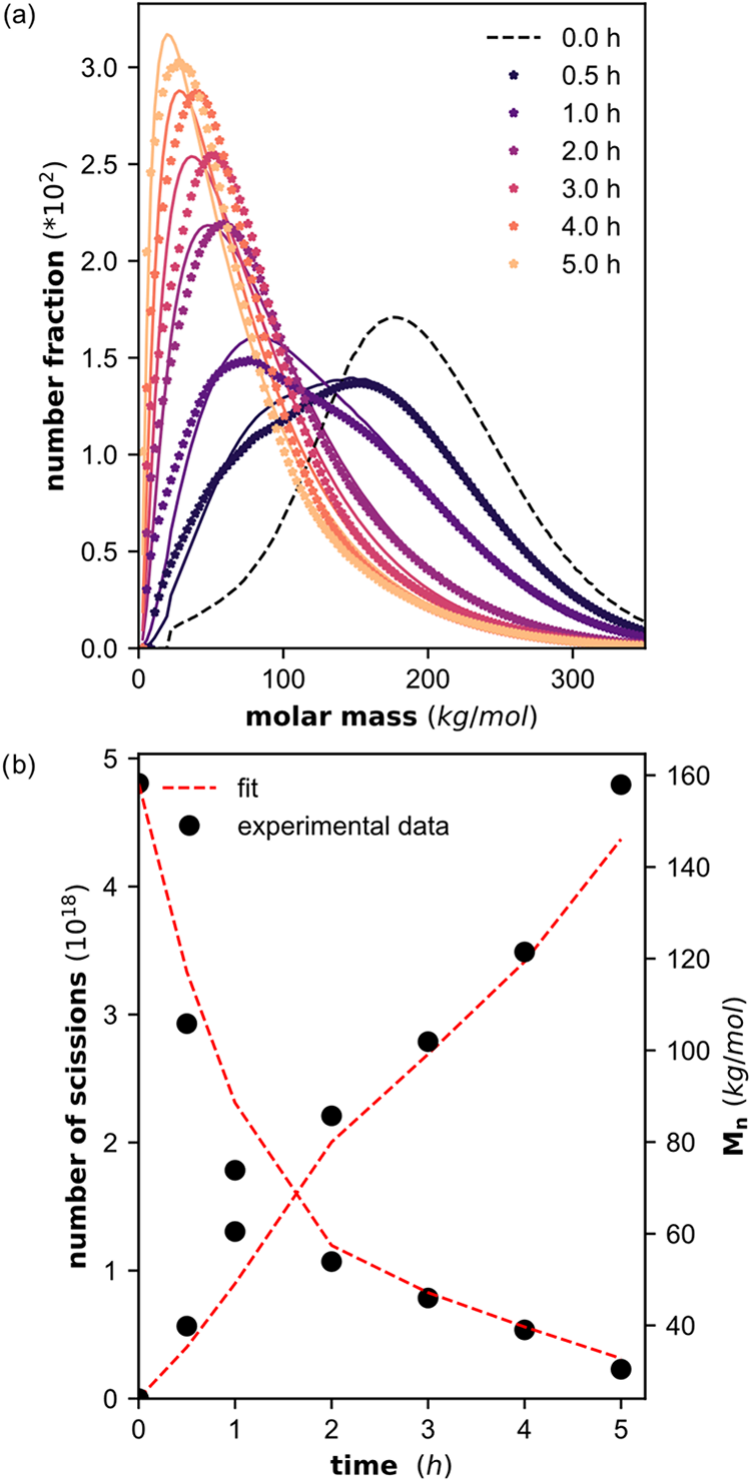
(a) Molar mass distributions (star symbols) obtained from HT-SEC before and after milling of 300 mg of PE_160_ for 0.5, 1, 2, 3, 4, and 5 h at 30 Hz at RT under air in a 25 mL steel container, using 5 steel grinding spheres (10 mm) together with fits (solid lines) obtained with the model using *N* = 30. *r* = 0.13 and *s* = 0.58 are obtained. (b) Number of scissions determined by eq 1 and number averaged molar mass over milling time together with values obtained from the fit.

Since the distribution of chain cleavage probability is intrinsically wider for longer chains (Figure S7b), it is misleading to say that the chain is cleaved “within the middle 15% of the chain” as stated previously for *r* = 0.15.^27^ Alternatively, one could state that there is a 68% probability that the chain is cleaved within the inner 30% of the chain. It is a very wide statistical distribution around the center. In addition, the fact that *r* does not depend on the milling conditions casts doubt on a physical origin but rather suggests that it is unlikely to cleave the polymer at the chain ends under any condition. This is because the chain ends are mobile and a mechanochemical cleavage requires some fixture of the chain at each side around the cleavage location. This is most likely deep in the chain. In this case, the polymer protrudes the longest from either side of the cleavage point and it is most likely that these ends are either fixed in a crystal or an entanglement (Scheme 2a). We will see below that entanglements are crucial to achieve polymer cleavage.

Interestingly, *r* is dependent on the degree of degradation that is fitted. This is most clearly illustrated when fitting each time point individually for the milling conditions in Figure 3. The fits become much better and *r* decreases with more cleavage and lower *M*_n_ values (Figure S9). We attempt to explain this by a critical minimum length *c* that is necessary to immobilize the polymer chain on both sides of the mechanochemical cleavage point, e.g., in crystallites or entanglements (Scheme 4). It is assumed that shorter segments can slip out of their fixation upon mechanical impact, so that the energy of the latter can be dissipated by chain movement without bond cleavage. Mechanochemical chain cleavage is especially unlikely close to dangling chain ends, because these segments are the most flexible.

**Scheme 4:**
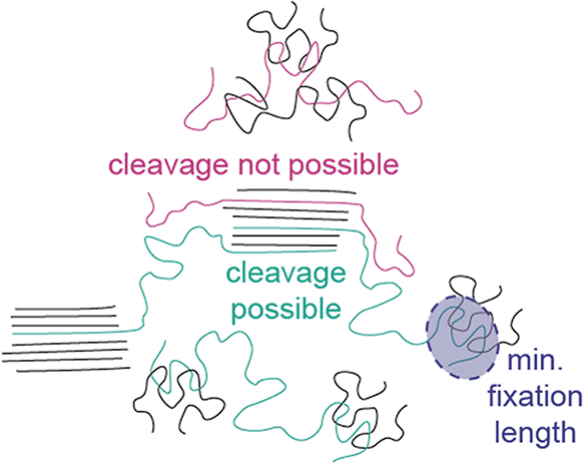
Illustration of Likely Cleavage between Fixture Points, Which Leads to A Statistical Cleavage Probability Centered around the Middle of the Chain

In a very simplified model, we simulate the effect of *c* on *r* by considering all possible positions of two fixation blocks on polymer chains of a given length *l*. The location of the two blocks is allowed to freely slide along the polymer chain, without the two blocks overlapping (Figure S28a). For the sake of simplicity, chain cleavage is assumed to occur exactly in the middle between the two blocks. This results in Δ-shaped distributions of cleavage probability centered around the middle of the chain (Figure S28b). The relative width of these distributions (*r*) correlates with the inverse chain length *l* (Figure S28c). Thus, shorter chains have a slightly higher selectivity for cleavage deep in the chain, which is in qualitative accordance with the observed lower *r* values for lower *M*_n_ (Figure S9). For the data of Figure S9, a critical minimum fixation length of *c* = 12.1 ± 2.6 kg/mol was calculated which corresponds to a PE chain segment with a length of 108 ± 23 nm. We recognize that this is quite a bit higher than the limiting molar mass obtained with an extruder previously, which could be due to the relatively mild conditions used for the data in Figure S9, namely milling at RT.

### Effect of Entanglement on Chain Cleavage

In addition to determining the cleavage location along the chain, we investigated the influence of entanglements on polymer cleavage. We milled an UHMWPE synthesized in a highly diluted aqueous emulsion polymerization. From this polymerization single chain PE (PE_single_) nanocrystals are obtained which cannot entangle during growth because of the compartmentalized character of the reaction and a fast crystallization compared to polymerization rate.^14^ The analysis of this sample with DSC and the effectiveness in creating entanglements upon annealing (PE_anneal_) is described in the SI, Section S6.^28^ Upon milling, the degree of crystallinity increased slightly for PE_anneal_, suggesting that more degradation occurs in the amorphous regions. For PE_single_ crystallinity decreased, suggesting that crystalline regions are also affected. While milling leads to some amorphization in the first few minutes, in many of the experiments with different PEs, the degree of crystallinity levels out at around 60% (Figure S31). Thus, we cannot exclude that some chain cleavage occurs in crystalline regions, however, the amorphous regions seem more affected.

Especially chains spanning over several crystals seem to be prone to degradation. PE_anneal_ experienced a significantly more pronounced decrease in *M*_n_ from 1566 to 84 kg/mol than the disentangled, single chain nanocrystal sample, which only decreased to 313 kg/mol (Figure 4). This is a remarkable difference, indicating the effective cleavage of entangled and tie chains.

**Figure 4:**
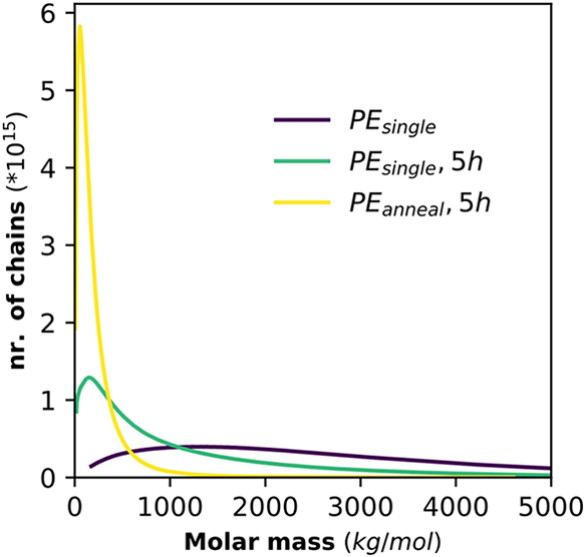
Molar mass distributions measured by HT-SEC of the untreated sample before milling and the untreated and annealed sample after milling at 30 Hz, at RT under N_2_ for 5 h using ZrO_2_ spheres.

## Conclusions

The synthesis of well-defined PE models with a narrow molar mass distribution allows for the systematic study of chain cleavage mechanisms in polyolefin materials that are much more complex than previously studied fully amorphous PS and PMMA. Polyolefins comprise both amorphous and crystalline domains. Our adaptation of the chain cleavage model by Glynn et al. enables the simultaneous fitting of HT-SEC data of samples obtained after various times of milling. This together with the well-defined materials enables the determination of the chain cleavage location along the chain. Cleavage probability is highest deep in the chain and lowest at the chain ends. The fitting parameters obtained are very similar for different conditions suggesting a statistical distribution of chain cleavage probability, because the polymer needs to be fixed by entanglements or a crystal on either side of the cleavage site for cleavage to occur and this is the most likely to occur deep inside the polymer chain. The importance of entanglements for cleavage was further illustrated by the significantly faster cleavage of an entangled polymer over its disentangled analog. However, a high crystallinity does not seem to be a prerequisite for cleavage nor is it decisive that the polymer is in a brittle state, i.e., below its glass transition temperature. The similarity in the cleavage probability distribution for milling under cryogenic conditions and at RT suggests that the faster decrease in molar mass observed in the former case is due to slower recombination kinetics at low temperature rather than the lack of stress dissipation in the glassy polymer. In addition, the presence of oxygen during milling leads to a stabilization of radicals and thus a faster decrease in *M*_n_. A similar stabilization effect was observed when milling with steel instead of zirconia spheres. A correlation of degradation rates with molar mass could not be found. These insight provide mechanistic understanding of mechanochemical chain cleavage for PE not previously studied in much depth and expands knowledge previously mostly obtained in ultrasonication or with fully amorphous polymers. The knowledge obtained here on PE is likely also applicable to PP, as both are partially crystalline and have a similar glass transition temperature. As these polymers make up more than 50% of the current global polymer production and are particularly dominant in packaging, the gained insights are useful for designing future mechanochemical strategies for chemical plastic recycling and can also be interesting to better understand unwanted degradation during mechanical recycling via extrusion. Understanding in the ball mill rather than ultrasonication is more industrially relevant as ball milling does not require solvent and is arguably more scalable.

## Supplementary Material



## Data Availability

This manuscript can also be found as executable paper via https://colab.research.google.com/github/InaVo/Chain-cleavage-Modelling-Paper/blob/main/Chain-Cleavage-Modelling.ipynb. The executable paper is set up as a jupyter notebook and includes all code used to analyze and plot the data and the raw data can also be found on the linked github repository.

## Notes

Calculations of kinetic parameters, additional NMR characterization, additional fits, simulation of fixation length, DSC analysis of UHMWPE (PDF)

## Abbreviations

BHT: di-*tert*-butyl hydroxytoluene
DSC: differential scanning calorimetry
HT-SEC: high temperature size exclusion chromatography
HT-NMR: high temperature nuclear magnetic resonance
MAO: methylaluminoxane
*M*_n_: number-averaged molar mass
*M*_w_: weight-average molar mass
PE: polyethylene
PEG: polyethylene glycol
PMMA: poly­(methyl methacrylate)
PP: polypropylene
PS: polystyrene
RT: room temperature
SDS: sodium dodecyl sulfate
TEMPO: 2,2,6,6-tetramethylpiperidinyloxyl
*T*_g_: glass transition temperature
UHMWPE: ultrahigh molecular weight PE
