# Supplementary material for: Modeling and Mechanistic Study of Polyethylene Chain Cleavage during Ball Milling
Source: Macromolecules. 2025 Oct 10;58(20):11388–96. doi: 10.1021/acs.macromol.5c02110 (PMC12573783; doi:10.1021/acs.macromol.5c02110)
Supplement: Supplementary file 1 [file ma5c02110_si_001.pdf]

Supporting information

CONTENTS

S1. Conditions for PE synthesis
S2. Radical trapping experiments
S3. Calculations of reaction rates
S4. HT-NMR results
S5. Chain cleavage modelling results
S6. Disentangled and annealed polymer and crystallinity

ABBREVIATIONS

BHT, di-*tert*-butyl hydroxytoluene; DSC, differential scanning calorimetry; HT-SEC, high temperature size exclusion chromatography; HT-NMR, high temperature nuclear magnetic resonance; MAO, methylaluminoxane; M_n, number averaged molar mass; M_w, weight average molar mass; PE, polyethylene; PEG, polyethylene glycol; PMMA, polymethylmethacrylate; PP, polypropylene; PS, polystyrene; RT, room temperature; SDS, sodium dodecyl sulfate; TEMPO, 2,2,6,6-tetramethylpiperidinyloxyl; T_g, glass transition temperature; UHMWPE, ultra-high molecular weight PE

S1. Conditions for PE synthesis

Table S1. Conditions for the PE synthesis. All experiments were performed at room temperature.

**Polymer notation**	**Cat. Amount** [µmol]	**Cat. Amount** [mg]	**MAO eq.** [-]	**MAO amount** [g]	**Reaction time** [min]	**Toluene volume** [ml]	**Stirring speed** [rpm]	**Yield** [g]	$\boldsymbol{M_n}$ [g/mol]	$\boldsymbol{M_w/M_n}$ [-]
**PE_{46}**	200	121.1	2000	23.2	5	700	500	n.d.	45,700	1.09
**PE_{56}**	90	54.5	1341	7.00	10	400	500	4.3	61,700	1.08
**PE_{103}**	60	36.3	100	0.35	8	300	1400	3.2	102,900	1.16
**PE_{160}**	30	18.2	2011	3.50	30	400	500	8.2	159,400	1.25
**PE_{170}**	100	60.6	2000	11.6	30	700	500	n.d.	169,800	1.10

S2. Radical trapping experiments

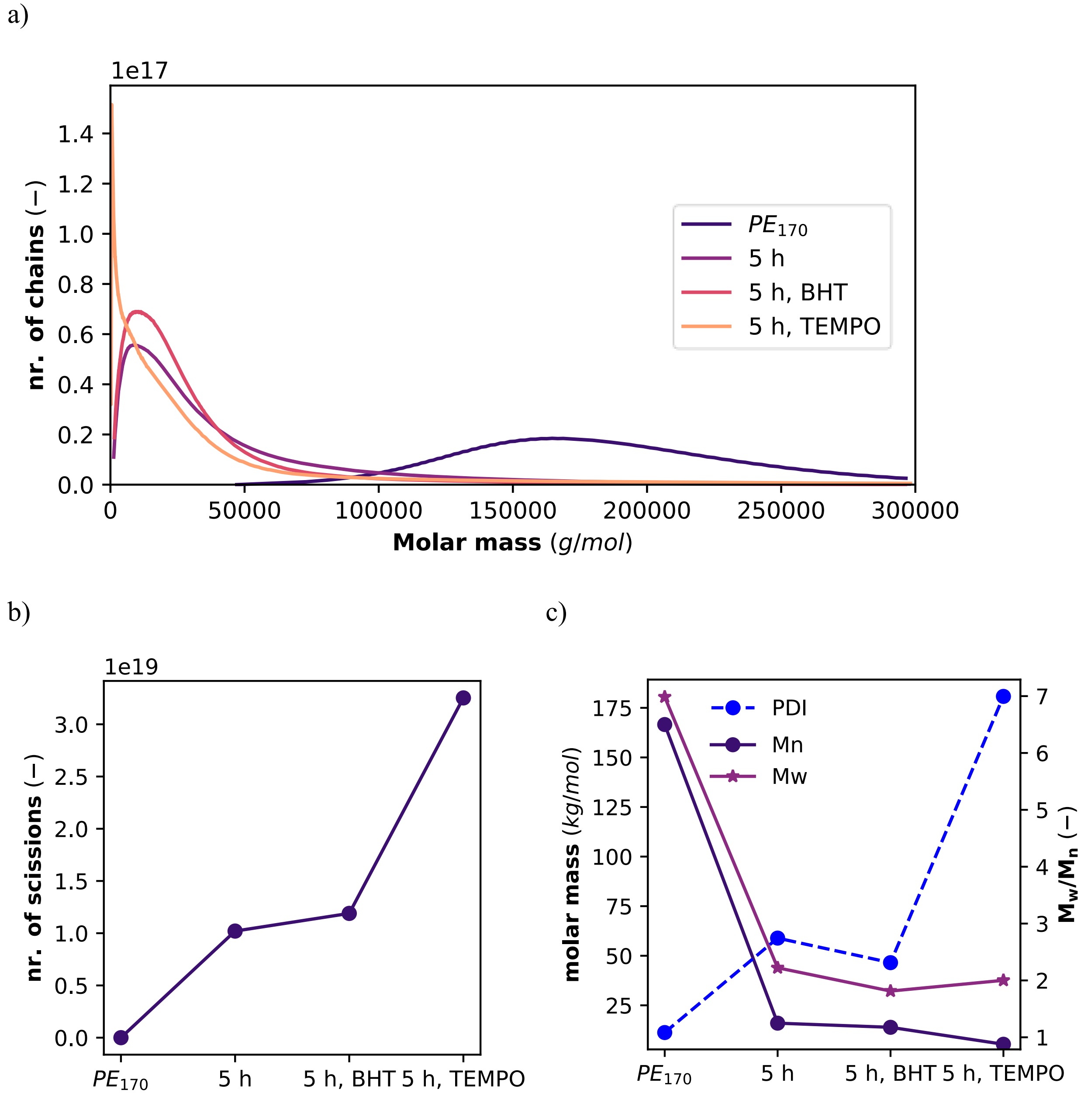

Figure S1. a) Molar mass distributions (solid lines) obtained from HT-SEC before and after milling of 300 mg of PE_{170} for 5 h at 30 Hz at cryogenic conditions under N_2 in a 25 ml steel container, using 5 steel grinding spheres (10 mm) adding either 30 mg of BHT or 21 mg of TEMPO. b) number of scissions, c) M_w, M_n and $\frac{M_w}{M_n}$ of the same samples as in a).

S3. Calculations of reaction rates

Reaction rate constants were calculated using Eq. S1 and kinetic constants taken from literature (**Table S1**).

$$k = A \cdot e^{-\frac{E_A}{RT}} \qquad \text{Equation S1}$$

Table S2. Pre-exponential factor and activation energy for disproportionation and recombination taken from Broadbelt et al.[11] as well as reaction rate constants calculated at 25 ˚C and -174.15 ˚C for recombination using Eq. S1.

	disproportionation	recombination	Reaction rate const. $\boldsymbol{k}$ at 25 ˚C [L mol s^{-1}]	Reaction rate const. $\boldsymbol{k}$ at -174.15 ˚C [L mol s^{-1}]
Pre-exponential factor $\boldsymbol{A}$ [L mol^{-1} s^{-1}]	$1.1 \cdot 10^{10}$	$1.1 \cdot 10^{11}$	$2.26 \cdot 10^{9}$	$1.03 \cdot 10^{6}$
Activation energy $\boldsymbol{E_A}$ [kcal mol^{-1}]	2.3			

S4. HT-NMR results

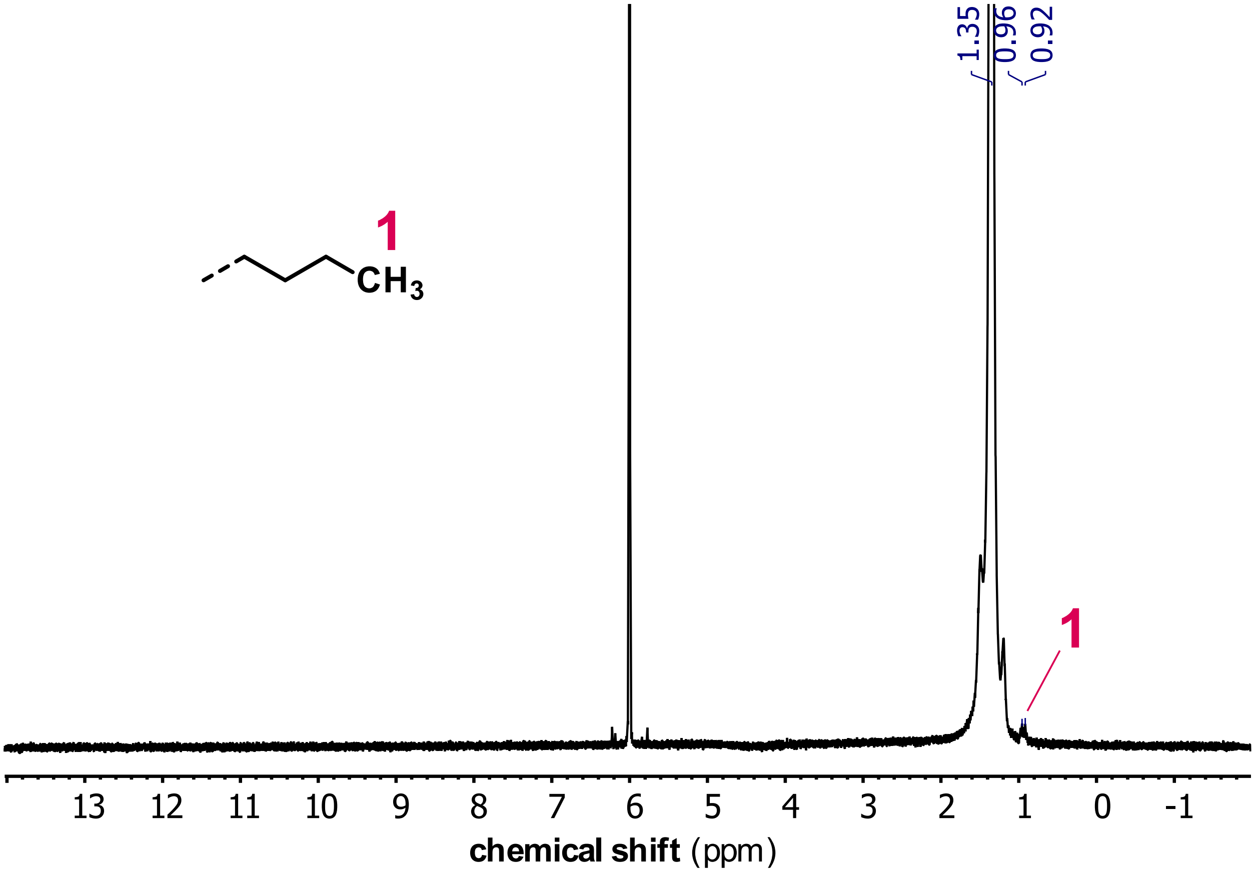

Figure S2. Exemplary ^{1}H NMR spectrum (400 MHz, 100 ˚C, $C_2D_2Cl_4$) of PE_{160} as obtained from the polymerization prior to milling experiments. In this case, no chlorinated backbone is observed due to a shorter high-temperature NMR measurement time.

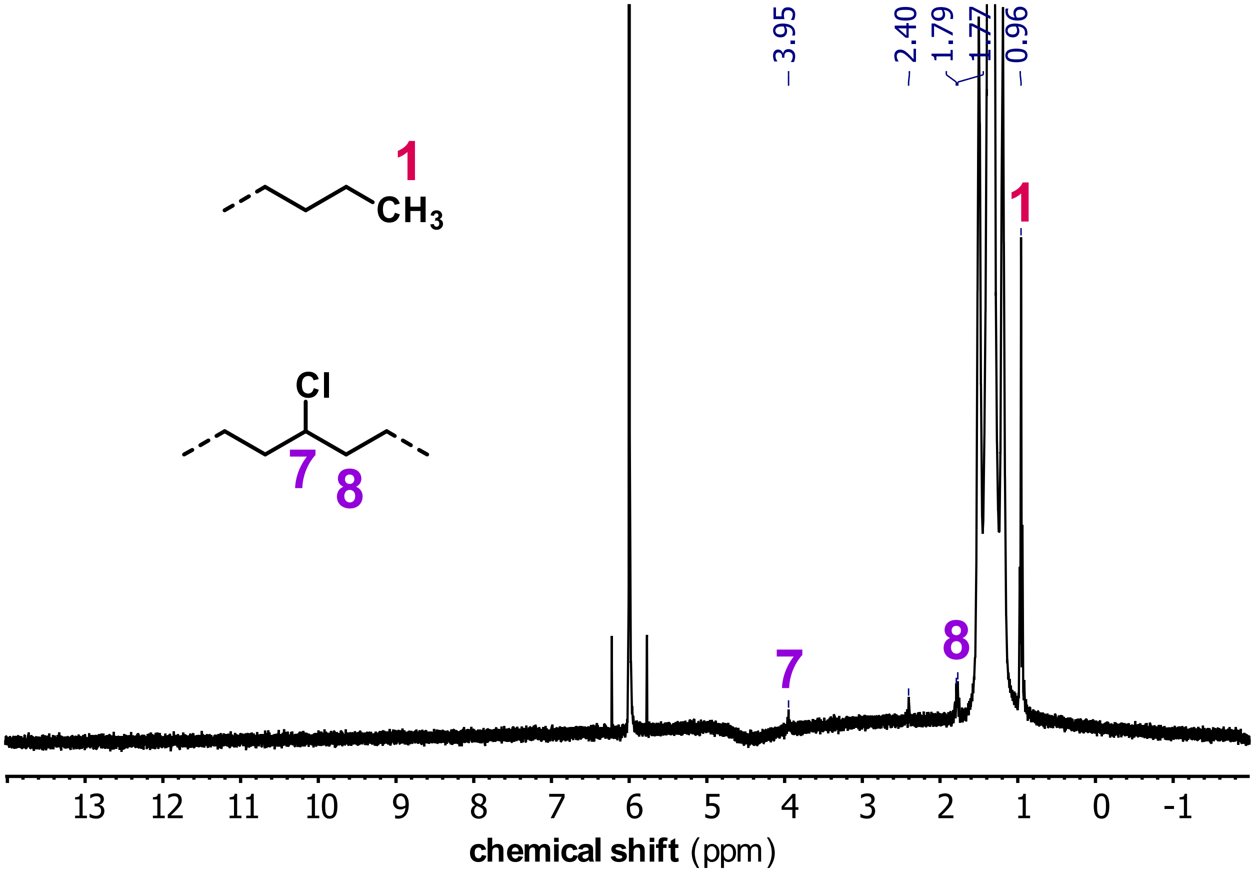

Figure S3. Exemplary ^{1}H NMR spectrum (400 MHz, 100 ˚C, $C_2D_2Cl_4$) of PE_{46} as obtained from the polymerization prior to milling experiments. The chlorinated PE backbone stems from reactions of the polymer with the NMR solvent during the long high-temperature measurements.

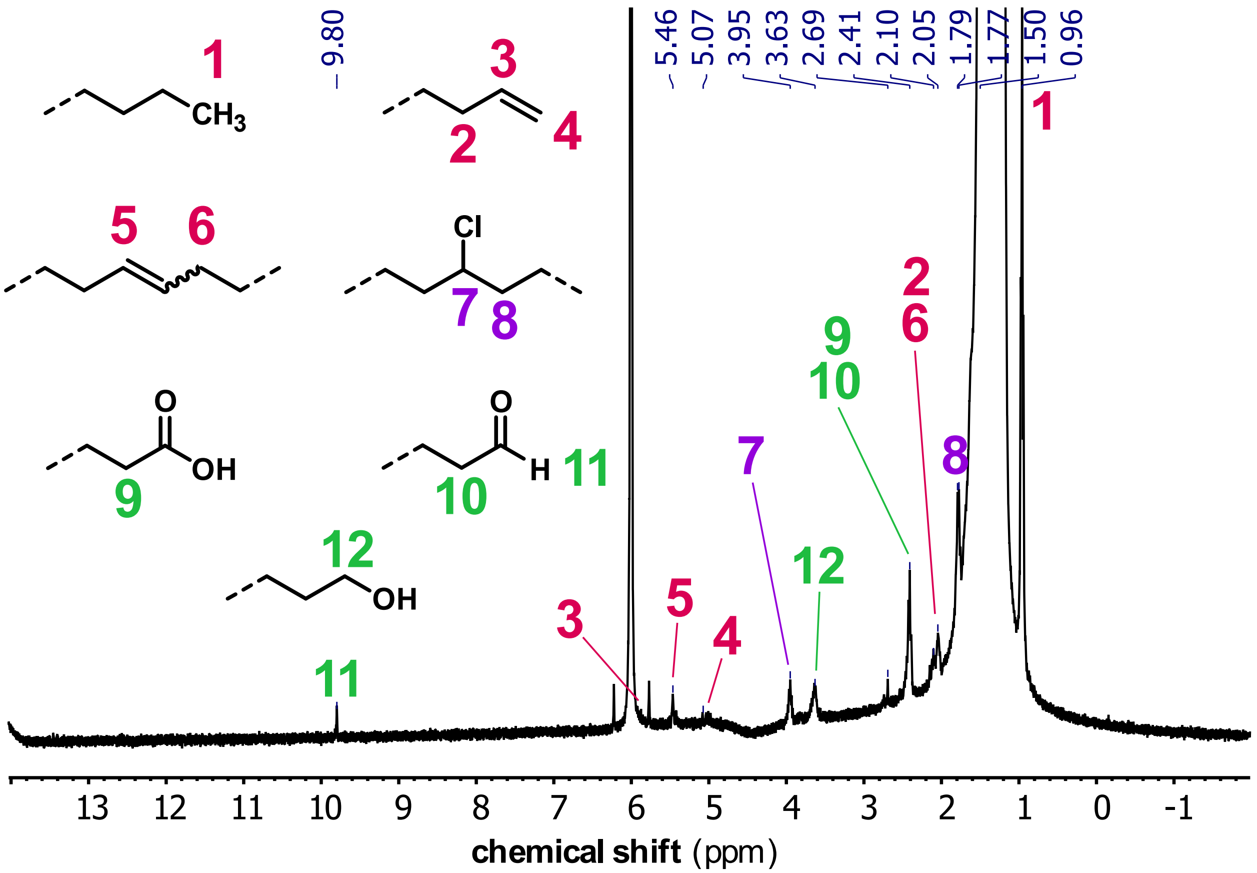

Figure S4. Exemplary ^{1}H NMR spectrum (400 MHz, 100 ˚C, $C_2D_2Cl_4$) of milled PE_{46} (milling conditions: 300 mg PE, 12 h, RT, neat oxygen, 5x zirconia grinding spheres with diameter of 10 mm). The chlorinated PE backbone stems from reactions of the polymer with the NMR solvent during the long high-temperature measurements.

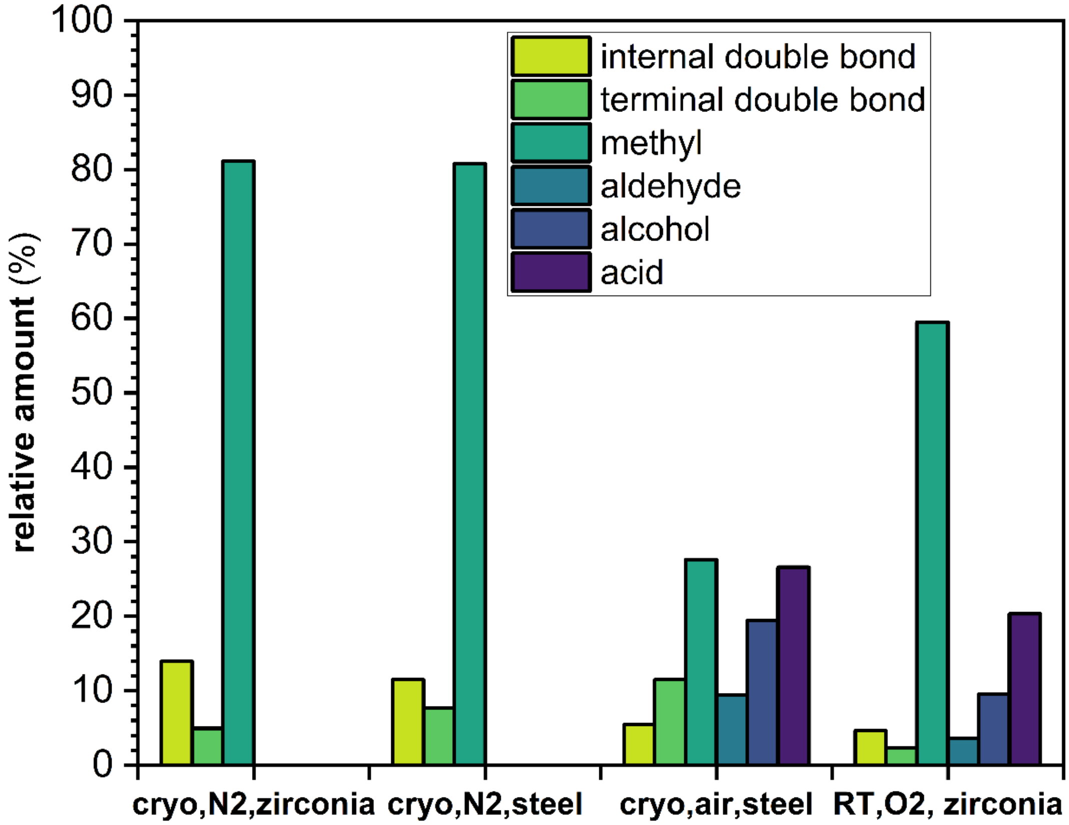

Figure S5. Relative amounts of functional groups in milled PE samples for different milling conditions as determined by ^{1}H NMR.

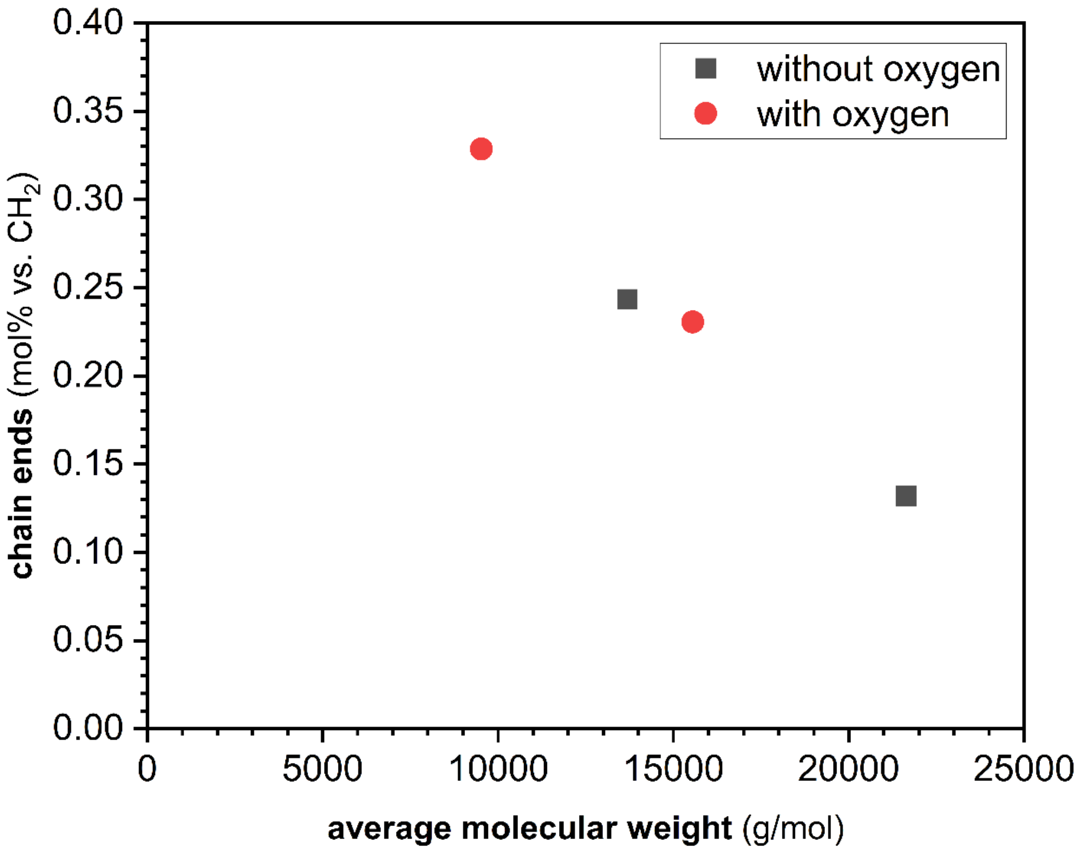

Figure S6. Correlation of number average molecular weight M_n as determined by HT-SEC with the total number of chain ends from ^{1}H NMR for PE milled under either N_2 or air. End groups in samples milled in the absence of oxygen are methyl and olefinic groups. Samples milled under air additionally contain aldehyde, alcohol and carboxylic acid end groups.

S5. Chain cleavage modelling results

In the first step of the model algorithm, certain chains are selected from the distribution, represented as a probability distribution curve (**Figure S7a**). This curve is calculated assuming that the probability of cleavage increases with molar mass (Eq. S2), which is the degree of polymerization x multiplied by the monomer molar mass $M_0 = 28\frac{g}{mol}$. The dependence is stronger for larger values of the exponent s. Eq. S2 is multiplied with the original molar mass distribution f_n to obtain the distribution curve of chains to select for cleavage (Eq. S3). At the first simulation step, the molar mass distribution of the original polymer, obtained from HT-SEC, is used as input.

$$C = (M_0 \cdot x)^s \qquad \text{Eq. S2}$$

$$P(t,x) = f_n \cdot C \qquad \text{Eq. S3}$$

The script iterates over the polymerization degree x and calculates $P(t,x)$. It also calculates how many chains of polymerization degree x are produced by cleavage of chains larger than x, starting from $y = x + 1$ (Eq. S4, **Figure S7b**).

$$\sum_{y=x+1}^{\infty} P(t,y)Q(y,x) \qquad \text{Eq. S4}$$

$$Q(y,x) = \frac{1}{ry\sqrt{2\pi}} e^{\left(-\frac{\left(x-\frac{y}{2}\right)^2}{2r^2y^2}\right)} \qquad \text{Eq. S5}$$

$P(t,y)$ is calculated the same way as $P(t,x)$ (Eq. S3) and $Q(y,x)$ is a Gaussian distribution centered around the polymerization degree $y/2$ for any polymer longer than the x of the current iteration (Eq. S5). Here, ry represents the width of the Gaussian distribution, equivalent to what is usually denoted as the standard deviation σ. A larger r represents a cleavage that is more broadly distributed around the center. However, this width of the distribution also increases with the degree of polymerization y (**Figure S7b)**. $Q(y,x)$ is calculated with x fixed and varying y and then multiplied by $P(t,y)$ to ensure that longer chains are preferably cleavage and taking the chain length distribution at each time step ($f_n(t,x)$) into account (**Figure S7c**).

At each simulation step t, the process is repeated by iteration over all x and a new chain length distribution is obtained via Eq. S6. Refer to Glynn *et al.* for the derivation.[17]

$$f_n(t+1,x) = \frac{(N+t)f_n(t,x) - P(t,x) + 2\left\{\sum_{y=x+1}^{\infty} P(t,y)Q(y,x)\right\}}{N+t+1} \qquad \text{Eq. S6}$$

Here N represents the number of chains, where in each simulation step a certain fraction of chains is cleaved, roughly scaling with $1/(N+t)$. N can be chosen freely as it does not have an influence on the final molar mass distribution. If N is large, the cleavage is milder with each step, while a small N leads to faster cleavage (**Figure S8a-d**). The resulting molar mass distribution with different choices of N are exemplified in **Figure S8e-f**.

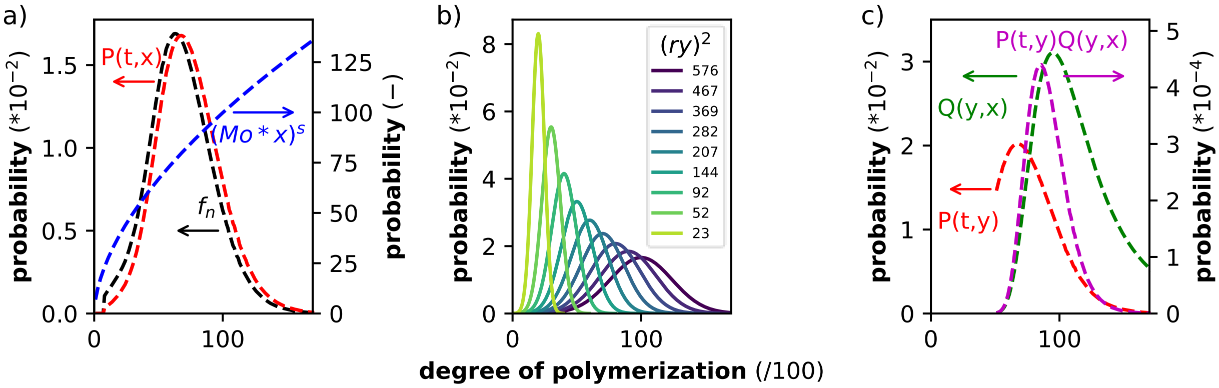

Figure S7. Illustration of the different steps in the simulation. a) Calculation of the distribution of chains to select for cleavage. b) Cleavage probability along the chain for chains with different degrees of polymerization. c) Illustration of $Q(y,x)$ calculated with x fixed at $x = 5000$ and as a function of y. The probability that a chain of length $y = 2x$ is broken is highest. In addition, the resulting curve after multiplication of $Q(y,x)$ by $P(t,y)$ is shown.

The chain cleavage probability is modelled with a Gaussian of a certain width ry. In our adaptation of the model, r is varied together with s to fit the measured molar mass distributions after certain milling times. If only one time point were to be fitted, N could simply be varied in addition to r and s to account for the degree of degradation. However, we aimed at fitting multiple time periods of milling simultaneously to obtain a more representative fit. Thus, N was fixed and the code chose the simulation steps that most closely represented each time point dynamically during the fitting procedure.

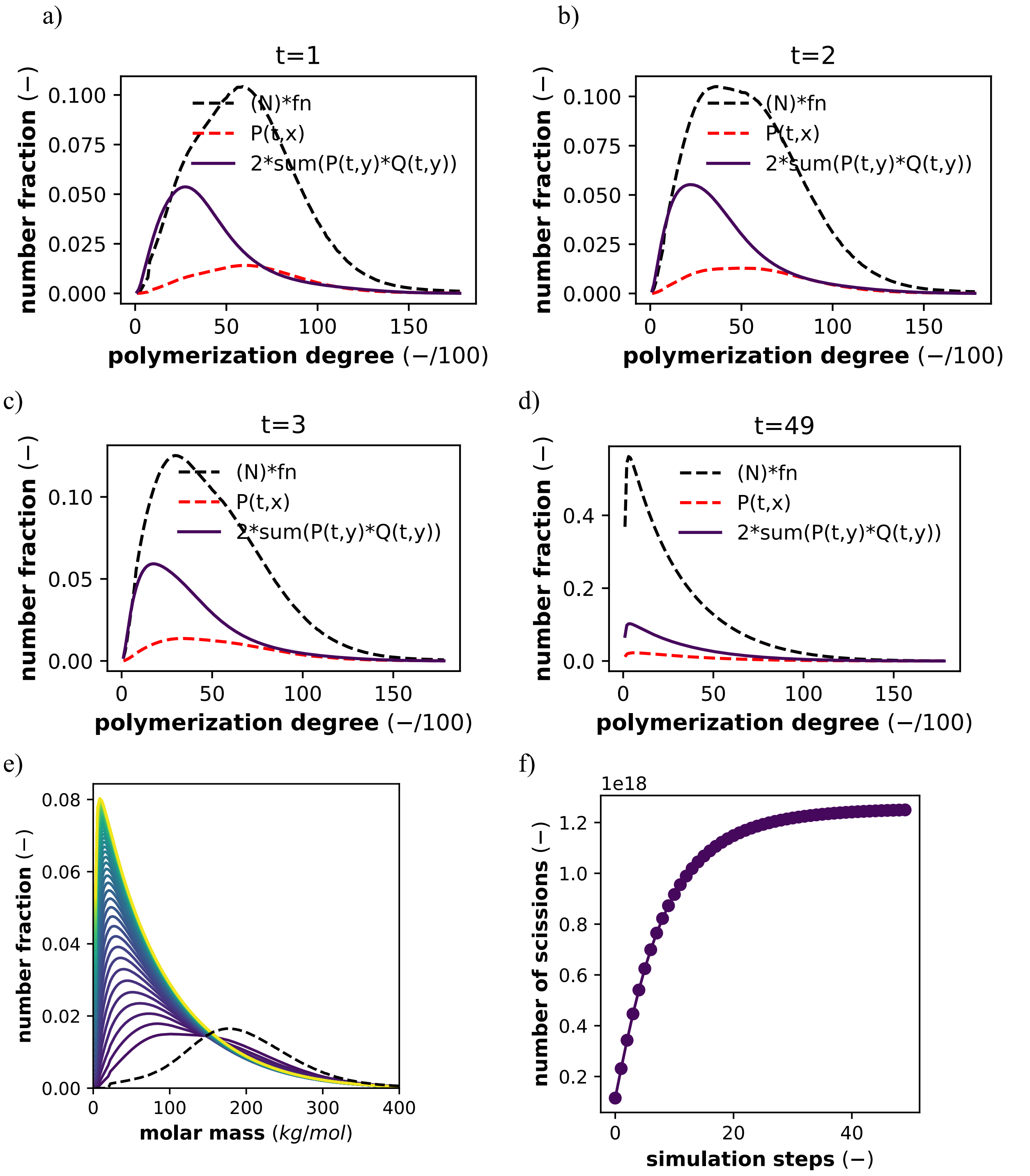

Figure S8. a)-d) Illustration of the original distribution, $P(t,x)$ (Eq. S3) and Eq. S4, here shown as continuous function of x. The area under Eq. S3 becomes smaller with increasing simulation time steps t because it scales with $1/(N+t)$. The simulation was performed using $r = 0.14$, $s = 0.18$ and $N = 7$ and PE_{160} as starting polymer. e) Simulated molar mass distributions over 50 simulation steps. f) Number of scissions calculated over the 50 time points using Eq. 1.

At very high degrees of degradation, the model struggles to represent the molar mass distribution (**Figure S12**). The model predicts much less cleavage events with longer simulation times t, because the fraction of polymers cleaved scales with $1/(N + t)$ (**Figure S29**). For example, the first hour of milling is represented by 15 steps, while 5 h milling requires 6999 instead of the 75 steps expected for a linear scaling (**Figure 3**). The parameter s, representing the dependence of cleavage probability on molar mass, varies widely and no trend with number of scissions could be found. This is another indication that the degradation rate is independent of the initial M_n.

Table S3. Fitting parameters obtained for all milling conditions. Fits are shown in **Figure S10-27**. $N = 30$ was used for all fits except for entry 11, for which $N = 500$ was used.

entry	Polymer notation	Temp.	Atmo.	sphere material	r [-]	s [-]	Times [h]
1	PE_{160}	RT	air	steel	0.13	0.58	[0, 0.5, 1, 2, 3, 4, 5]
2		RT	air	ZrO_2	0.14	0.48	[0, 5]
3		RT	N_2	ZrO_2	0.13	0.24	[0, 0.5, 1, 2, 3, 4, 5]
4*		Cryo	air	steel	0.03	0.08	[0, 0.25, 0.5, 1, 5]
5		Cryo	air	steel	0.15	0.06	[0, 0.25, 0.5, 1]
6*		Cryo	N_2	steel	0.05	0.12	[0, 1, 5]
7		Cryo	N_2	steel	0.15	0.34	[0, 1]
8*		Cryo	N_2	ZrO_2	0.09	0.07	[0, 1, 5]
9	PE_{170}	RT	N_2	ZrO_2	0.15	0.04	[0, 0.5, 1, 2, 3, 4, 5]
10*		Cryo	N_2	steel	0.07	0.12	[0, 5]
11	PE_{56}	RT	N_2	ZrO_2	0.23	9.33	[0, 0.5, 1, 2, 3, 4, 5]
12		RT	air	ZrO_2	0.20	0.00	[0, 5]
13	PE_{46}	RT	N_2	ZrO_2	0.18	0.07	[0, 5, 12, 44]
14		RT	air	ZrO_2	0.18	0.00	[0, 5, 12]
15*	PE_{103}	RT	air	ZrO_2	0.10	0.25	[0, 1, 5]
16		RT	N_2	ZrO_2	0.15	0.00	[0, 5]
17		RT	air	steel	0.11	0.00	[0, 5]
28		RT	N_2	steel	0.12	0.00	[0, 5]

*obtained fit is poor.

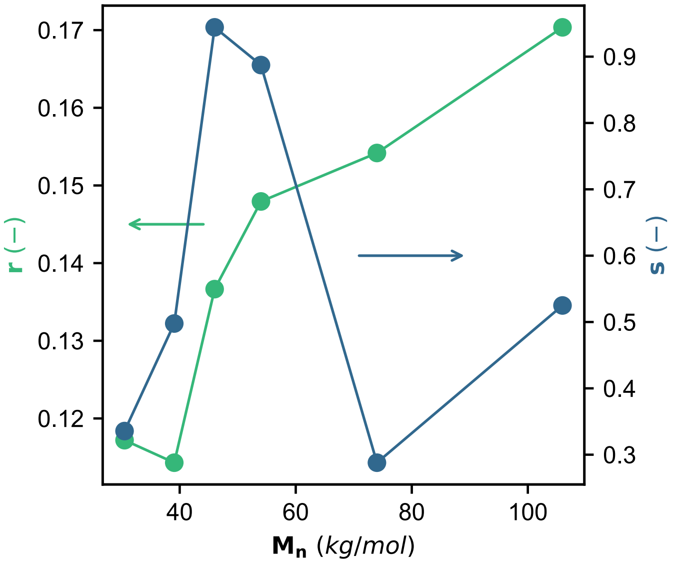

Figure S9. r and s values obtained with fitting each time point individually using $N = 30$ as a function of number of scissions calculated at this time point for milling of 300 mg of PE_{160} for 0.5, 1, 2, 3, 4 and 5 h at 30 Hz at RT under air in a 25 ml steel container, using 5 steel grinding spheres (10 mm).

Milling PE_{160}-Room Temperature

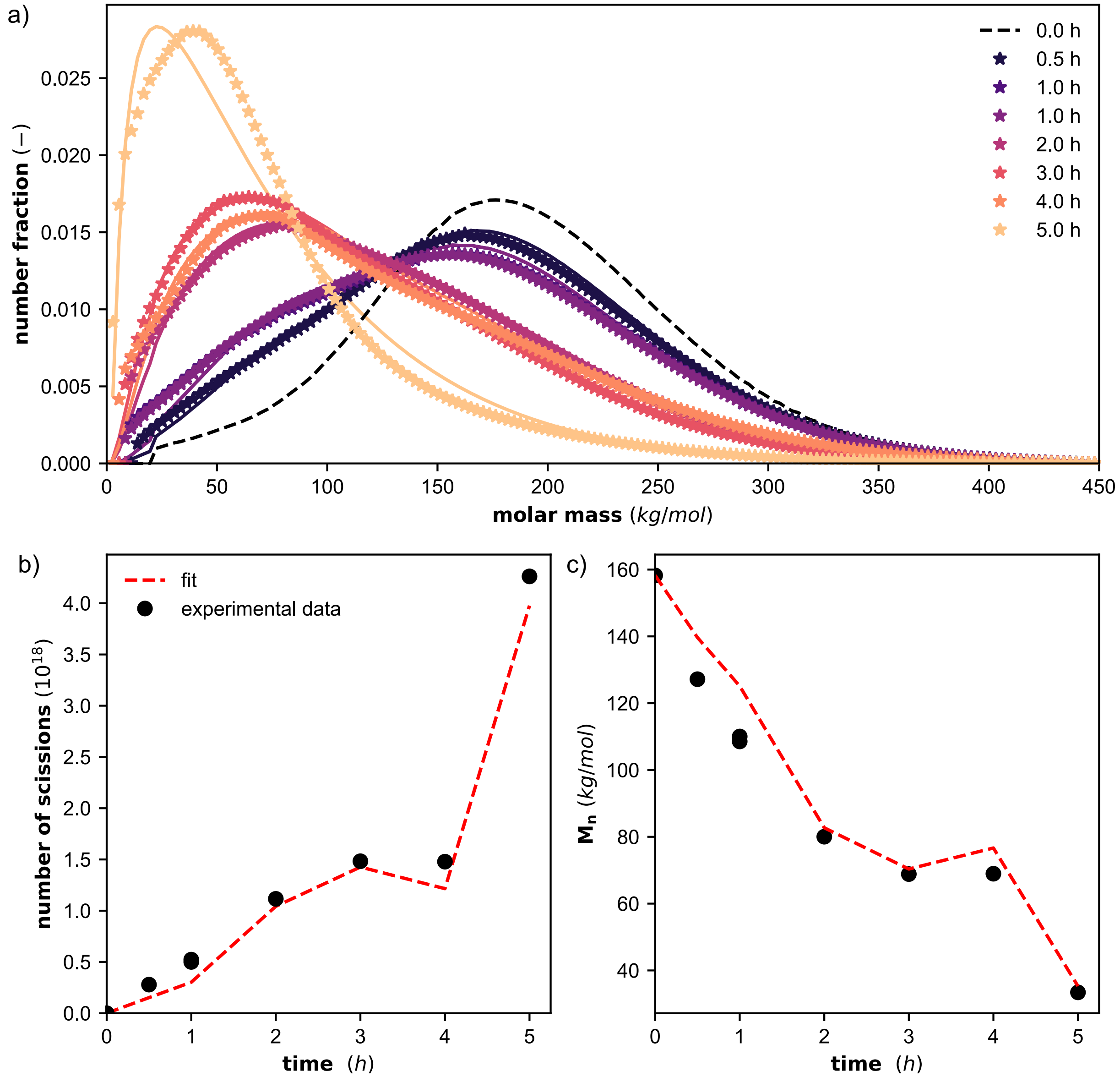

Figure S10. a) Molar mass distributions (solid lines) obtained from HT-SEC before and after milling of 300 mg of PE_{160} for 0.5, 1, 2, 3, 4 and 5 h at 30 Hz at RT under N_2 in a 25 ml steel container, using 5 ZrO_2 grinding spheres (10 mm) together with fits (star symbols) obtained with the model using $N = 30$. $r = 0.13$ $s = 0.24$ are obtained. b) Number of scissions determined by Eq. 1 and number averaged molar mass over milling time.

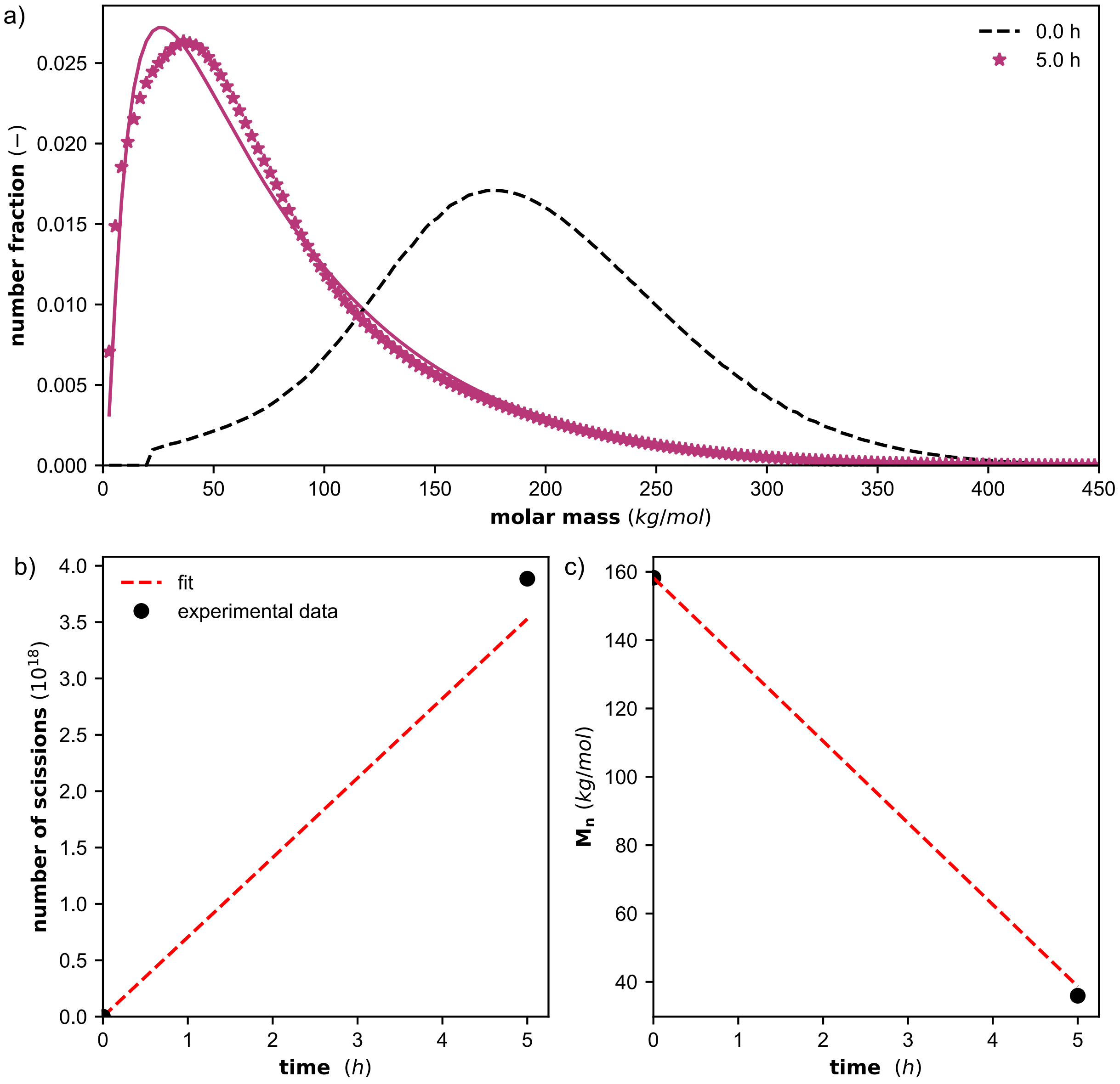

Figure S11. a) Molar mass distributions (solid lines) obtained from HT-SEC before and after milling of 300 mg of PE_{160} for 5 h at 30 Hz at RT under air in a 25 ml steel container, using 5 ZrO_2 grinding spheres (10 mm) together with fits (star symbols) obtained with the model using $N = 30$. $r = 0.14$ $s = 0.48$ are obtained. b) Number of scissions determined by Eq. 1 and number averaged molar mass over milling time.

Cryogenic conditions

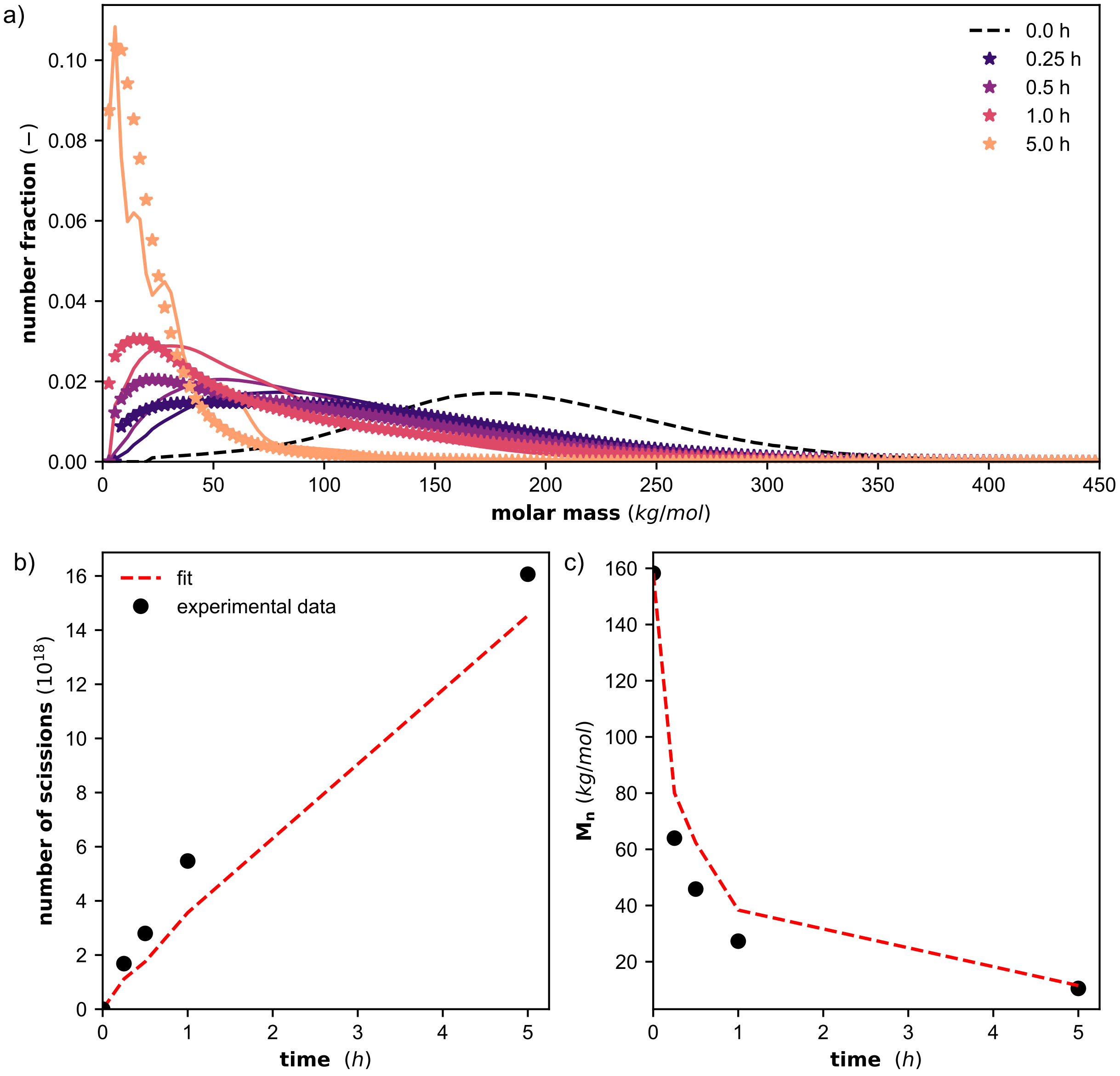

Figure S12. a) Molar mass distributions (solid lines) obtained from HT-SEC before and after milling of 300 mg of PE_{160} for 0.25, 0.5, 1, and 5 h at 30 Hz at cryogenic conditions under air in a 25 ml steel container, using 5 steel grinding spheres (10 mm) together with fits (star symbols) obtained with the model using $N = 30$. $r = 0.03$ $s = 0.08$ are obtained. b) Number of scissions determined by Eq. 1 and c) number averaged molar mass over milling time together with fits.

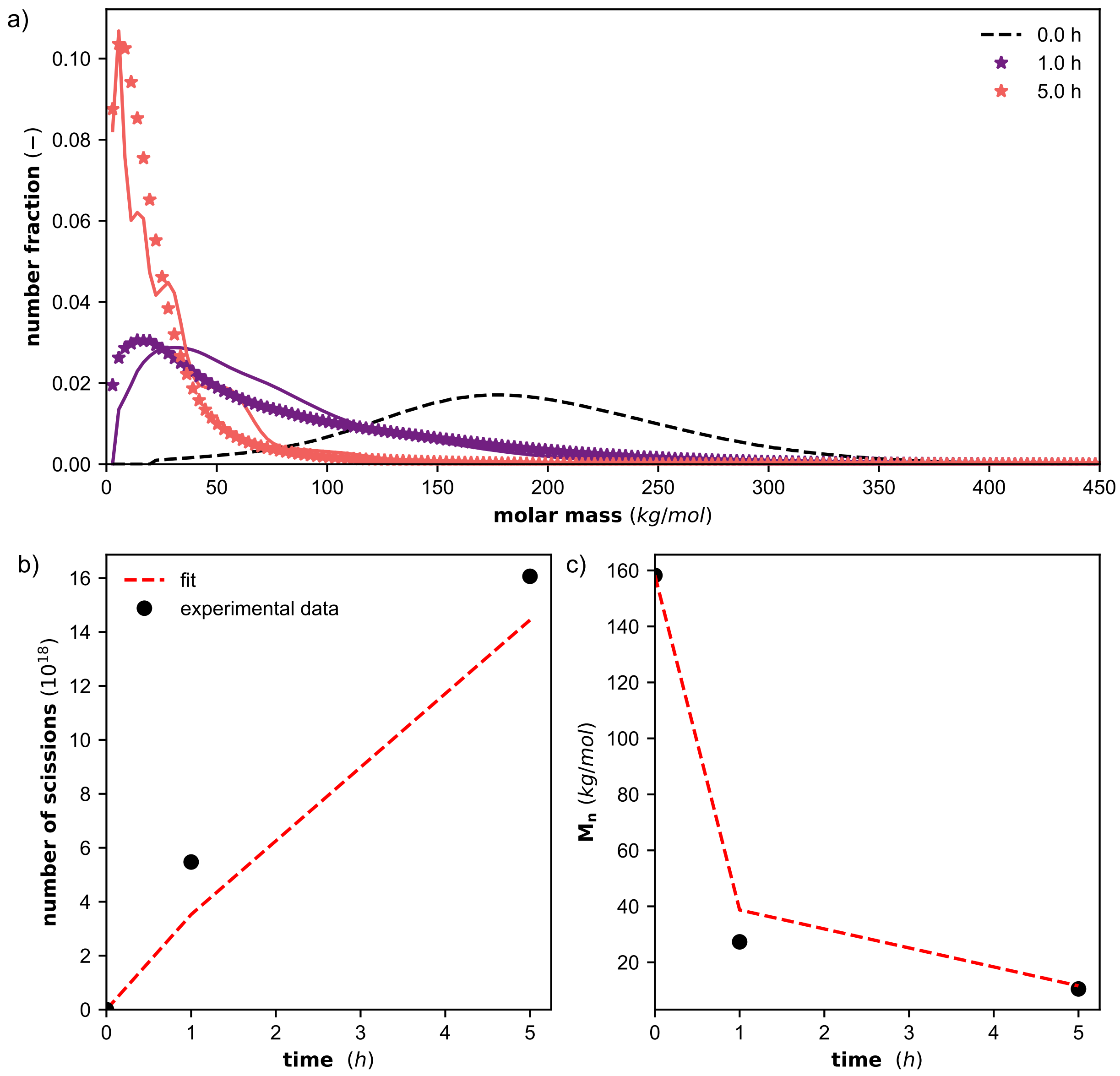

Figure S13. a) Molar mass distributions (solid lines) obtained from HT-SEC before and after milling of 300 mg of PE_{160} for 1, and 5 h at 30 Hz at cryogenic conditions under air in a 25 ml steel container, using 5 steel grinding spheres (10 mm) together with fits (star symbols) obtained with the model using $N = 30$. $r = 0.03$ $s = 0.09$ are obtained. b) Number of scissions determined by Eq. 1 and c) number averaged molar mass over milling time together with fits.

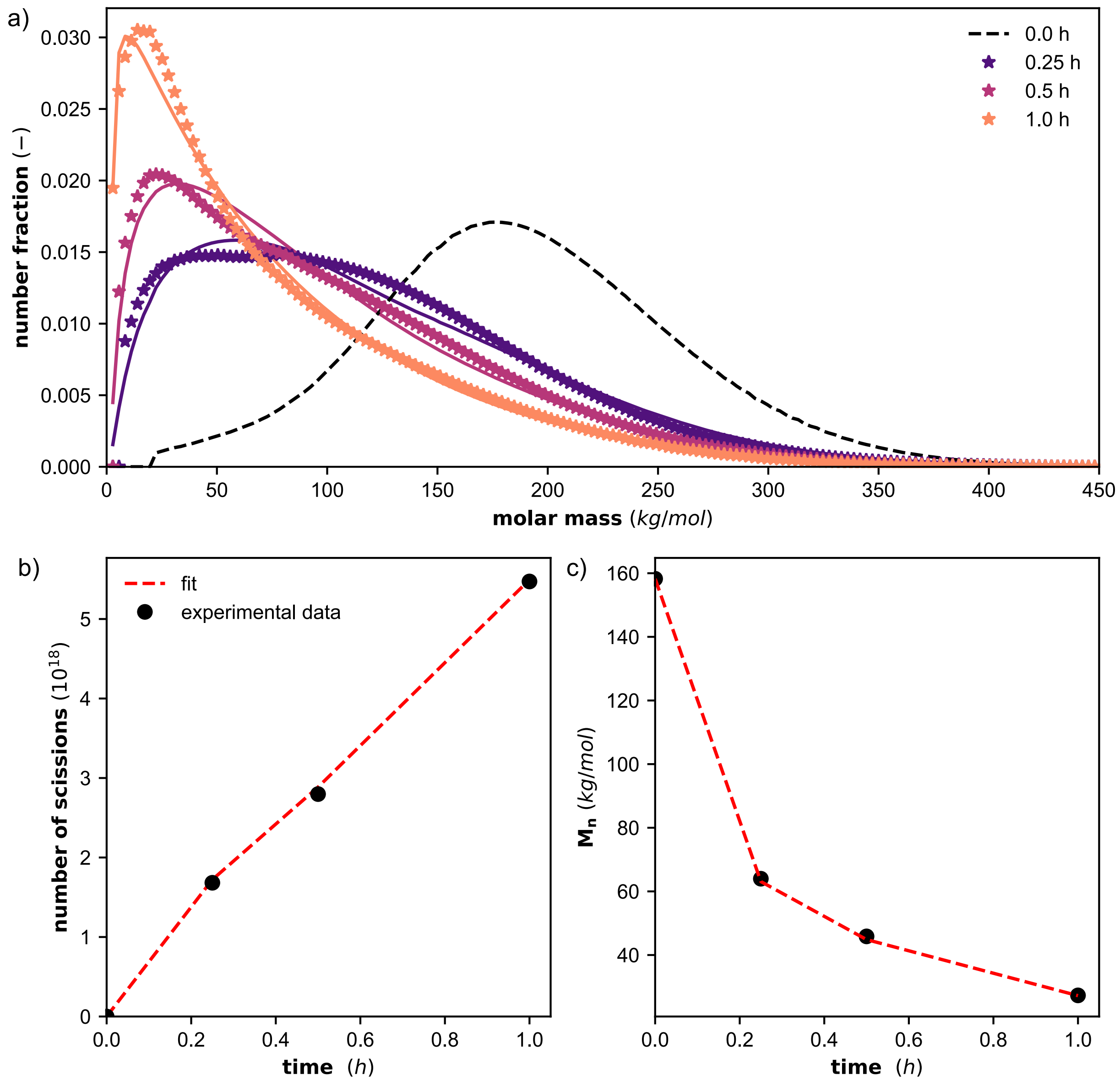

Figure S14. a) Molar mass distributions (solid lines) obtained from HT-SEC before and after milling of 300 mg of PE_{160} for 0.25, 0.5, and 1 h at 30 Hz at cryogenic conditions under air in a 25 ml steel container, using 5 steel grinding spheres (10 mm) together with fits (star symbols) obtained with the model using $N = 30$. $r = 0.15$ $s = 0.06$ are obtained. b) Number of scissions determined by Eq. 1 and c) number averaged molar mass over milling time together with results obtained from the fits.

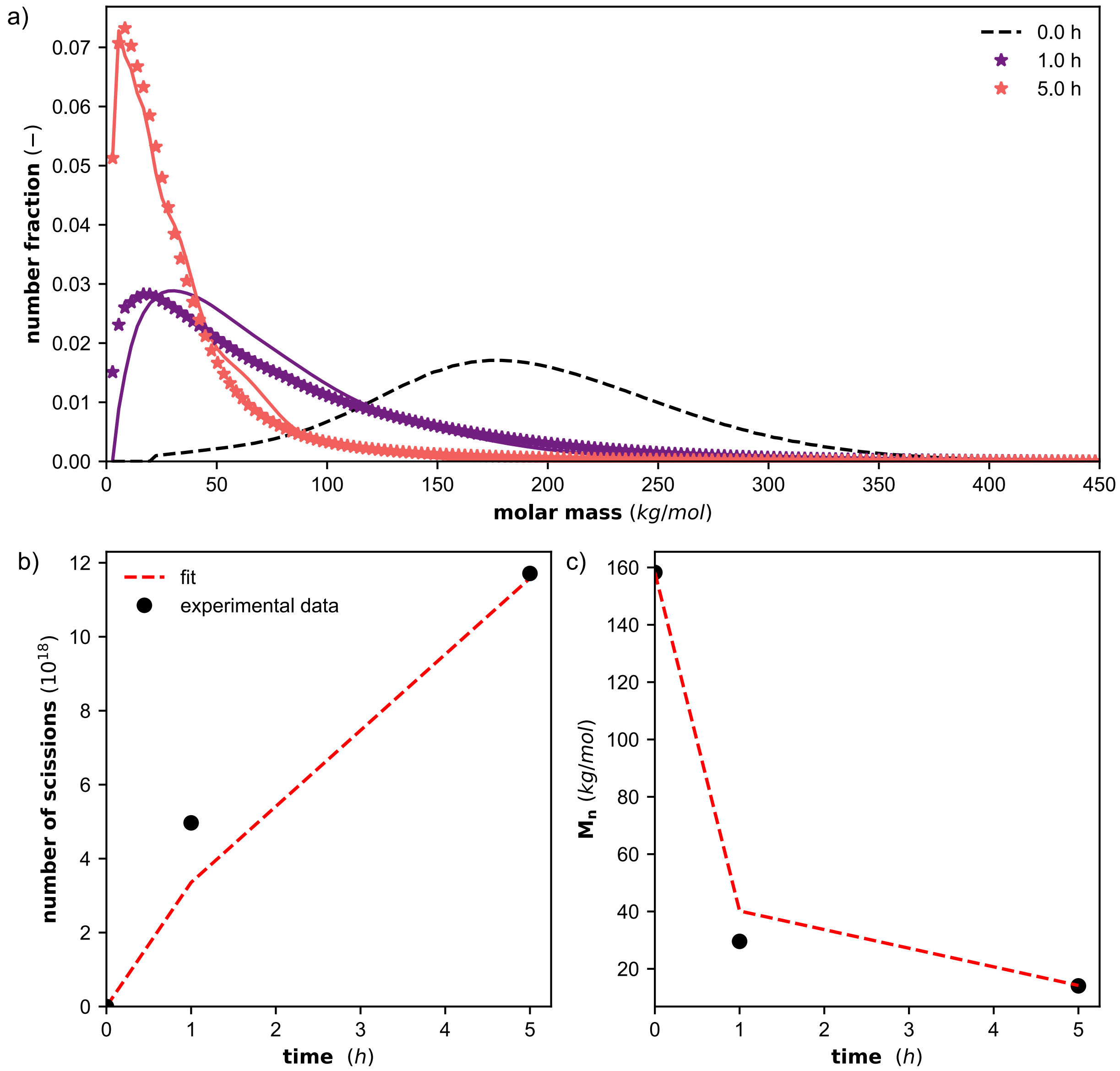

Figure S15. a) Molar mass distributions (solid lines) obtained from HT-SEC before and after milling of 300 mg of PE_{160} for 1 and 5 h at 30 Hz at cryogenic conditions under N_2 in a 25 ml steel container, using 5 steel grinding spheres (10 mm) together with fits (star symbols) obtained with the model using $N = 30$. $r = 0.05$ $s = 0.12$ are obtained. b) Number of scissions determined by Eq. 1 and c) number averaged molar mass over milling time together with values obtained from the fits.

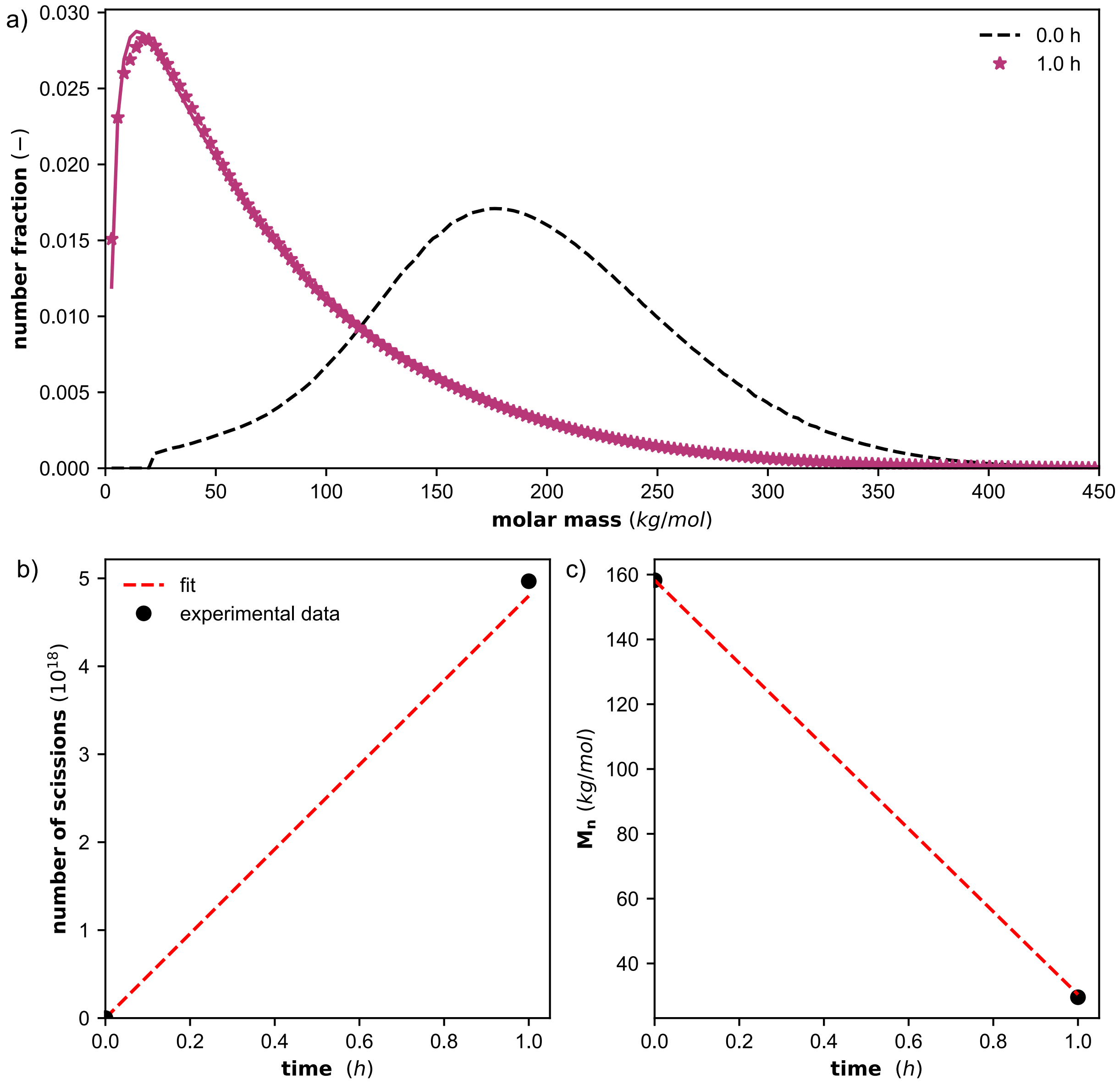

Figure S16. a) Molar mass distributions (solid lines) obtained from HT-SEC before and after milling of 300 mg of PE_{160} for 1 h at 30 Hz at cryogenic conditions under N_2 in a 25 ml steel container, using 5 steel grinding spheres (10 mm) together with fits (star symbols) obtained with the model using $N = 30$, $r = 0.15$ $s = 0.34$ are obtained. b) Number of scissions determined by Eq. 1 and c) number averaged molar mass over milling time together with values obtained from the fits.

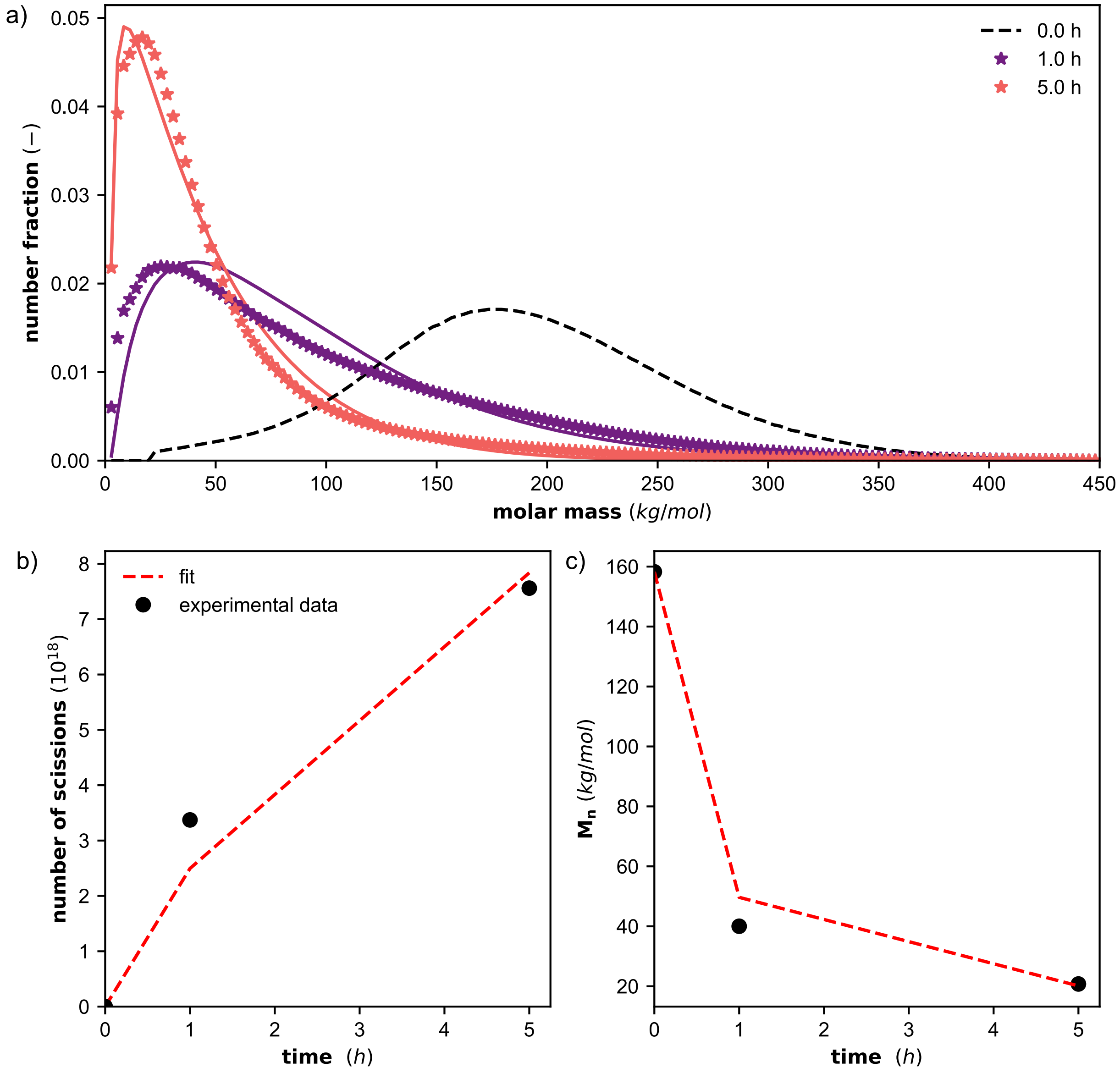

Figure S17. a) Molar mass distributions (solid lines) obtained from HT-SEC before and after milling of 300 mg of PE_{160} for 1 and 5 h at 30 Hz at cryogenic conditions under N_2 in a 25 ml steel container, using 5 ZrO_2 grinding spheres (10 mm) together with fits (star symbols) obtained with the model using $N = 30$. $r = 0.09$ $s = 0.07$ are obtained. b) Number of scissions determined by Eq. 1 and c) number averaged molar mass over milling time together with values obtained from the fits.

Milling PE_{170}

Room temperature

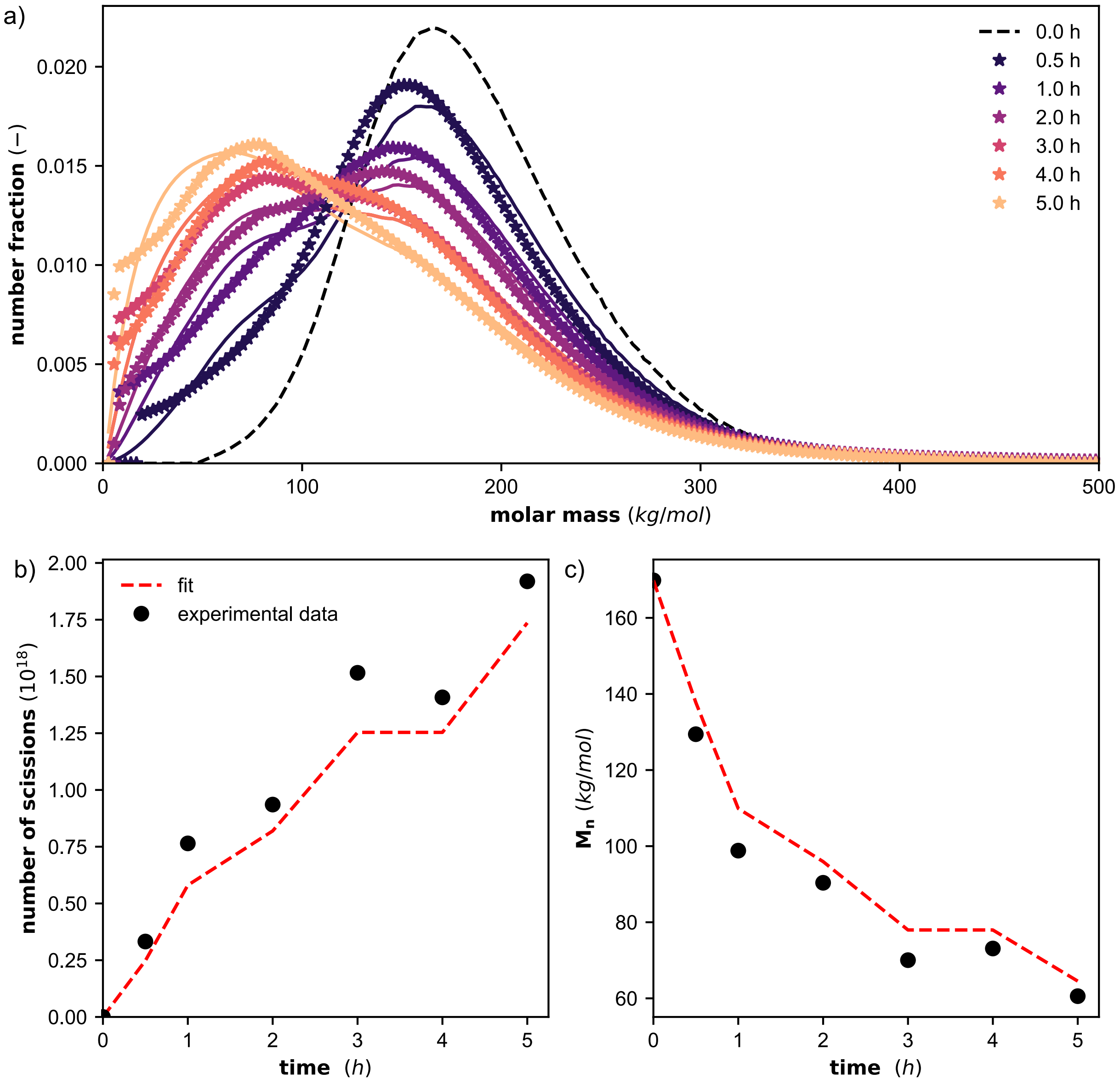

Figure S18. a) Molar mass distributions (solid lines) obtained from HT-SEC before and after milling of 300 mg of PE_{170} for 0.5, 1, 2, 3, 4 and 5 h at 30 Hz at RT under N_2 in a 25 ml steel container, using 5 ZrO_2 grinding spheres (10 mm) together with fits (star symbols) obtained with the model using $N = 30$. $r = 0.15$ $s = 0.04$ are obtained. b) Number of scissions determined by Eq. 1 and c) number averaged molar mass over milling time together with values obtained from the fits.

Cryogenic conditions

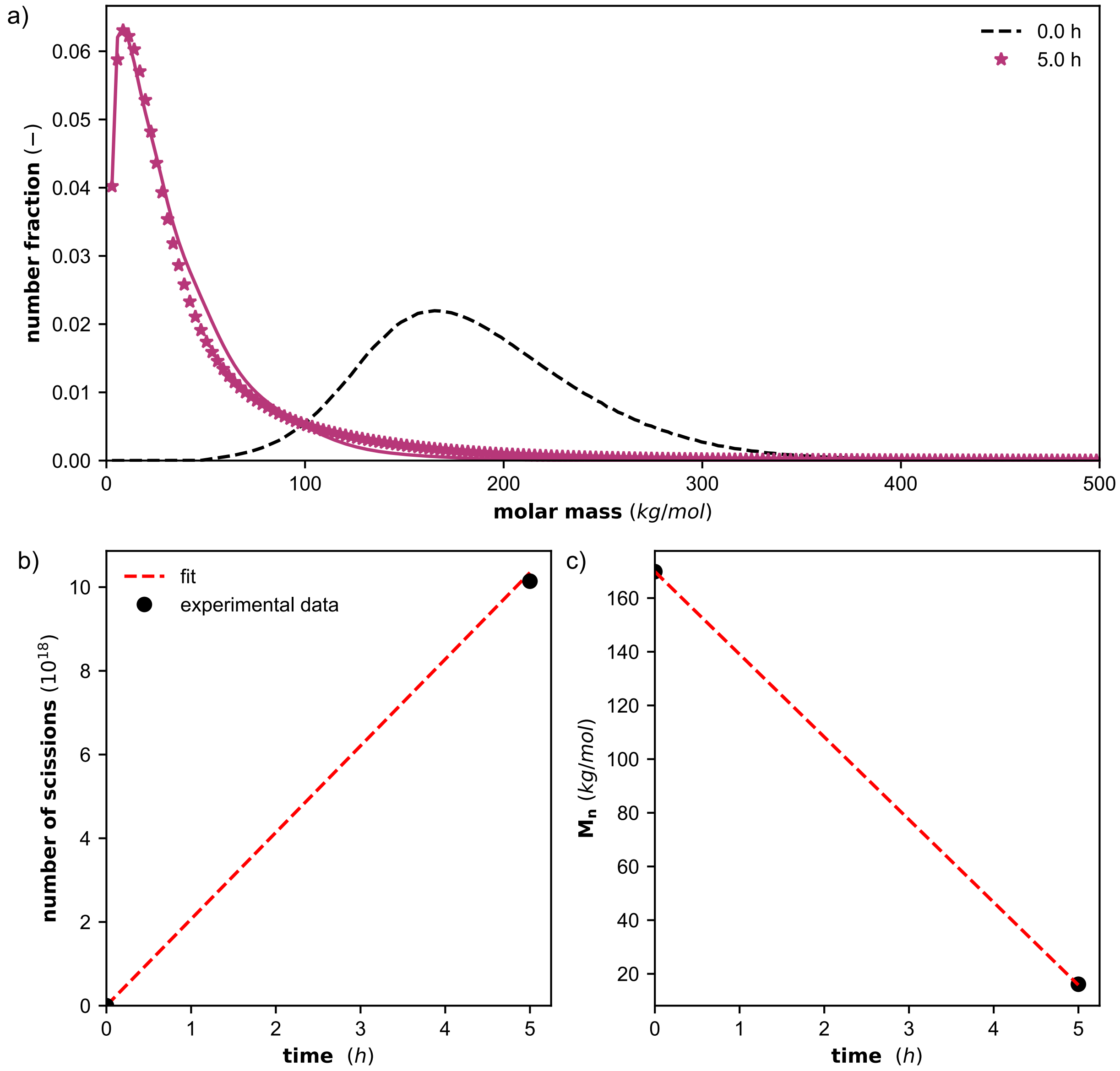

Figure S19. a) Molar mass distributions (solid lines) obtained from HT-SEC before and after milling of 300 mg of PE_{170} for 5 h at 30 Hz at cryogenic under N_2 in a 25 ml steel container, using 5 steel grinding spheres (10 mm) together with fits (star symbols) obtained with the model using $N = 30$. $r = 0.10$ $s = 0.14$ are obtained. b) Number of scissions determined by Eq. 1 and c) number averaged molar mass over milling time together with values obtained from the fits.

Milling PE_{56}

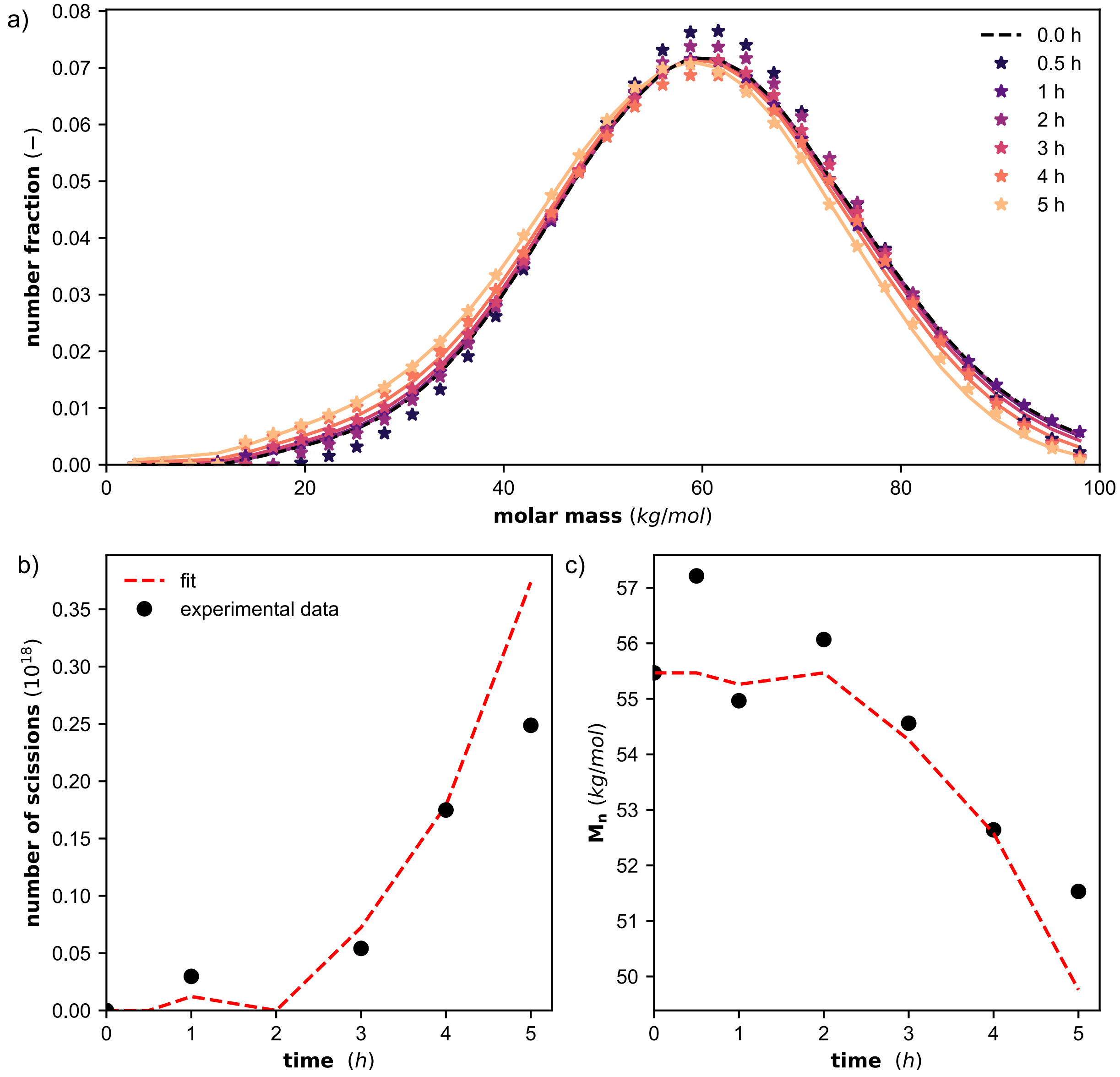

Figure S20. a) Molar mass distributions (solid lines) obtained from HT-SEC before and after milling of 300 mg of PE_{56} for 0.5, 1, 2, 3, 4, 5 h at 30 Hz at RT under N_2 in a 25 ml steel container, using 5 ZrO_2 grinding spheres (10 mm) together with fits (star symbols) obtained with the model using $N = 500$. . $r = 0.23$ $s = 9.33$ were obtained. No good fits could be obtained with $N = 30$ due to the low extent of chain cleavage with this polymer. b) Number of scissions determined by Eq. 1 and c) number averaged molar mass over milling time together with values obtained from the fits.

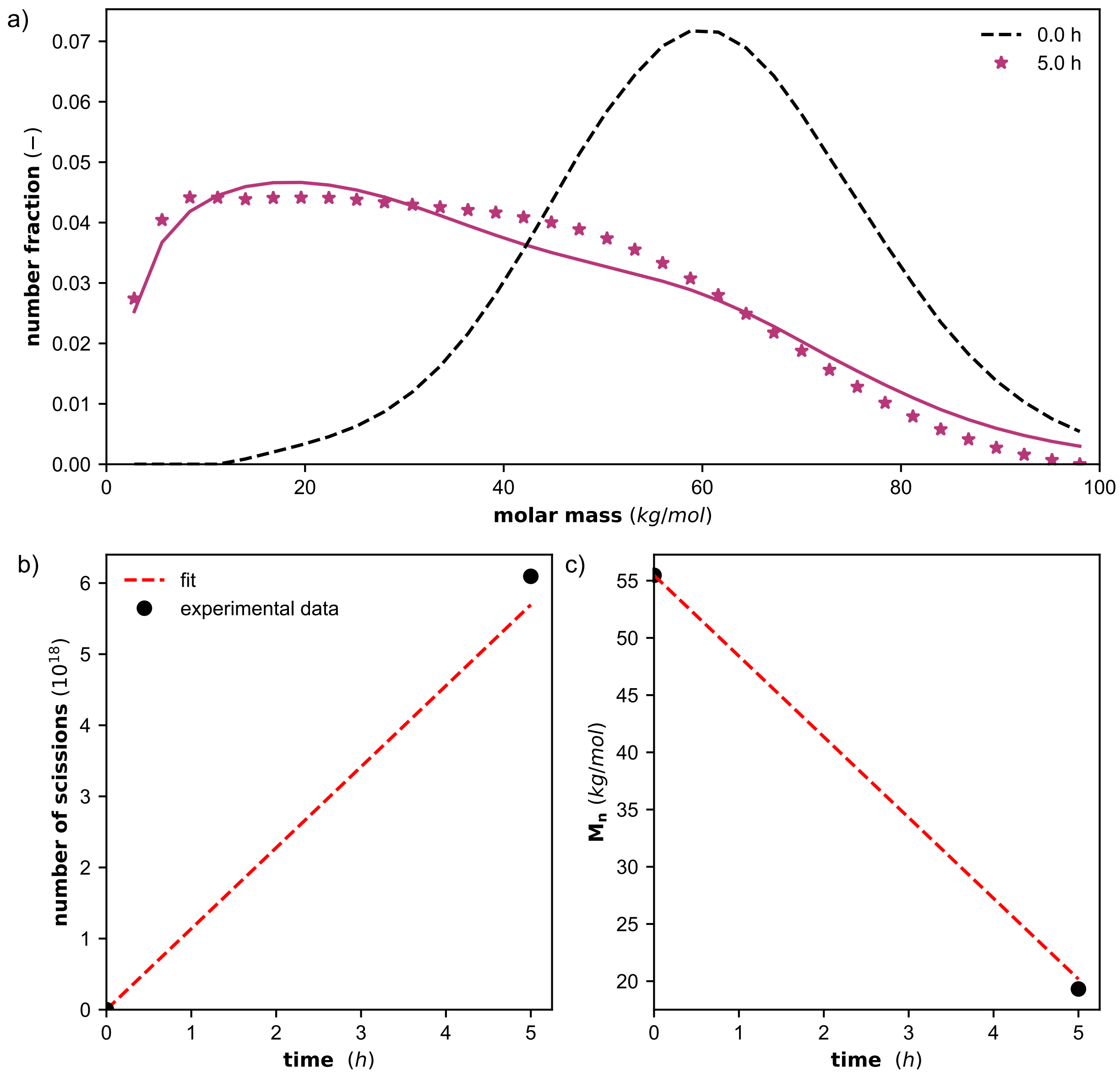

Figure S21. a) Molar mass distributions (solid lines) obtained from HT-SEC before and after milling of 300 mg of PE_{56} for 5 h at 30 Hz at RT under air in a 25 ml steel container, using 5 ZrO_2 grinding spheres (10 mm) together with fits (star symbols) obtained with the model using $N = 30$. $r = 0.20$ $s = 0.00$ were obtained. b) Number of scissions determined by Eq. 1 and c) number averaged molar mass over milling time together with values obtained from the fits.

Milling PE_{46}

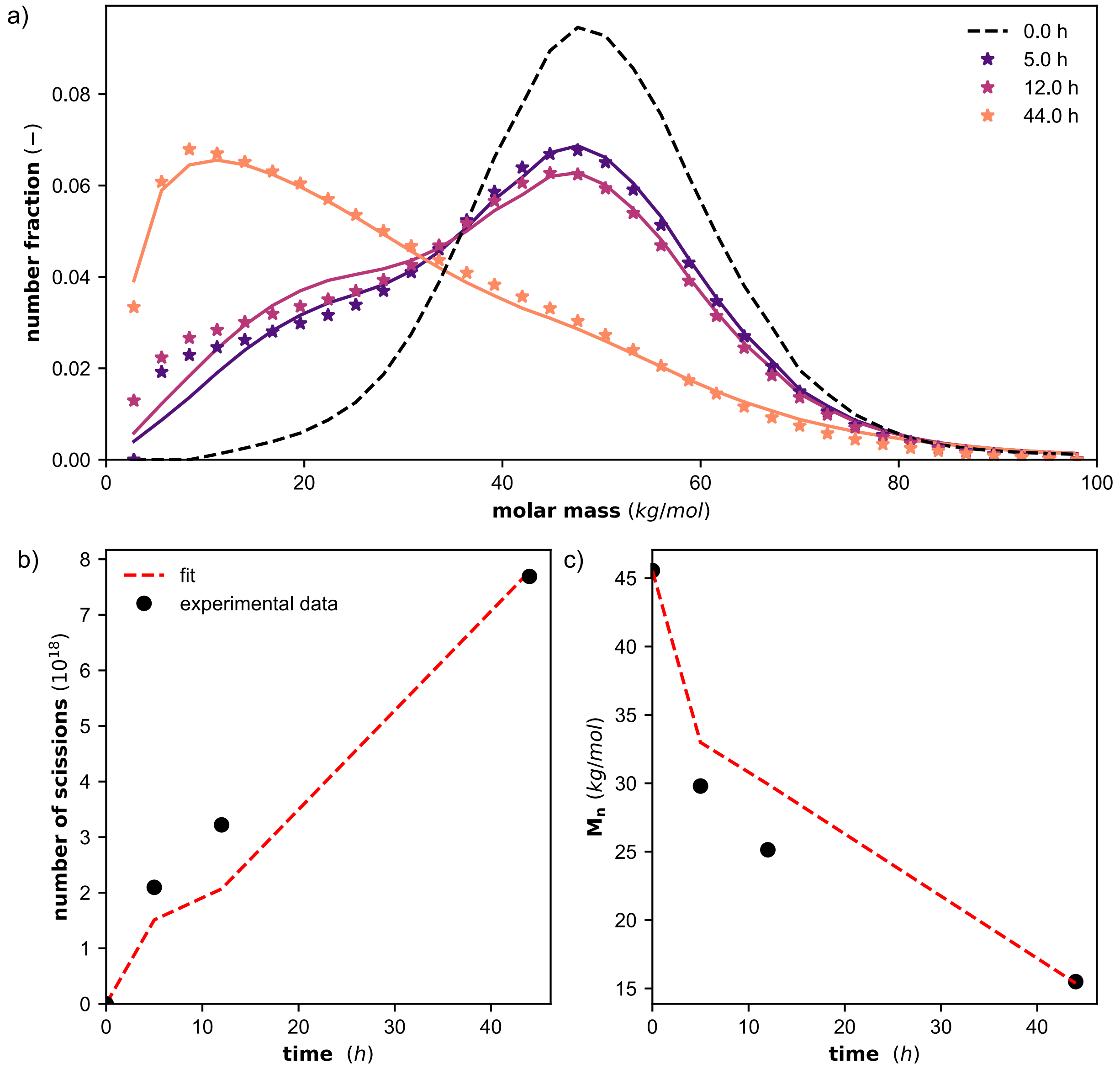

Figure S22. a) Molar mass distributions (solid lines) obtained from HT-SEC before and after milling of 300 mg of PE_{46} for 5, 12 and 44 h at 30 Hz at RT under N_2 in a 25 ml steel container, using 5 ZrO_2 grinding spheres (10 mm) together with fits (star symbols) obtained with the model using $N = 30$. $r = 0.18$ $s = 0.07$ are obtained. b) Number of scissions determined by Eq. 1 and c) number averaged molar mass over milling time together with values obtained from the fits.

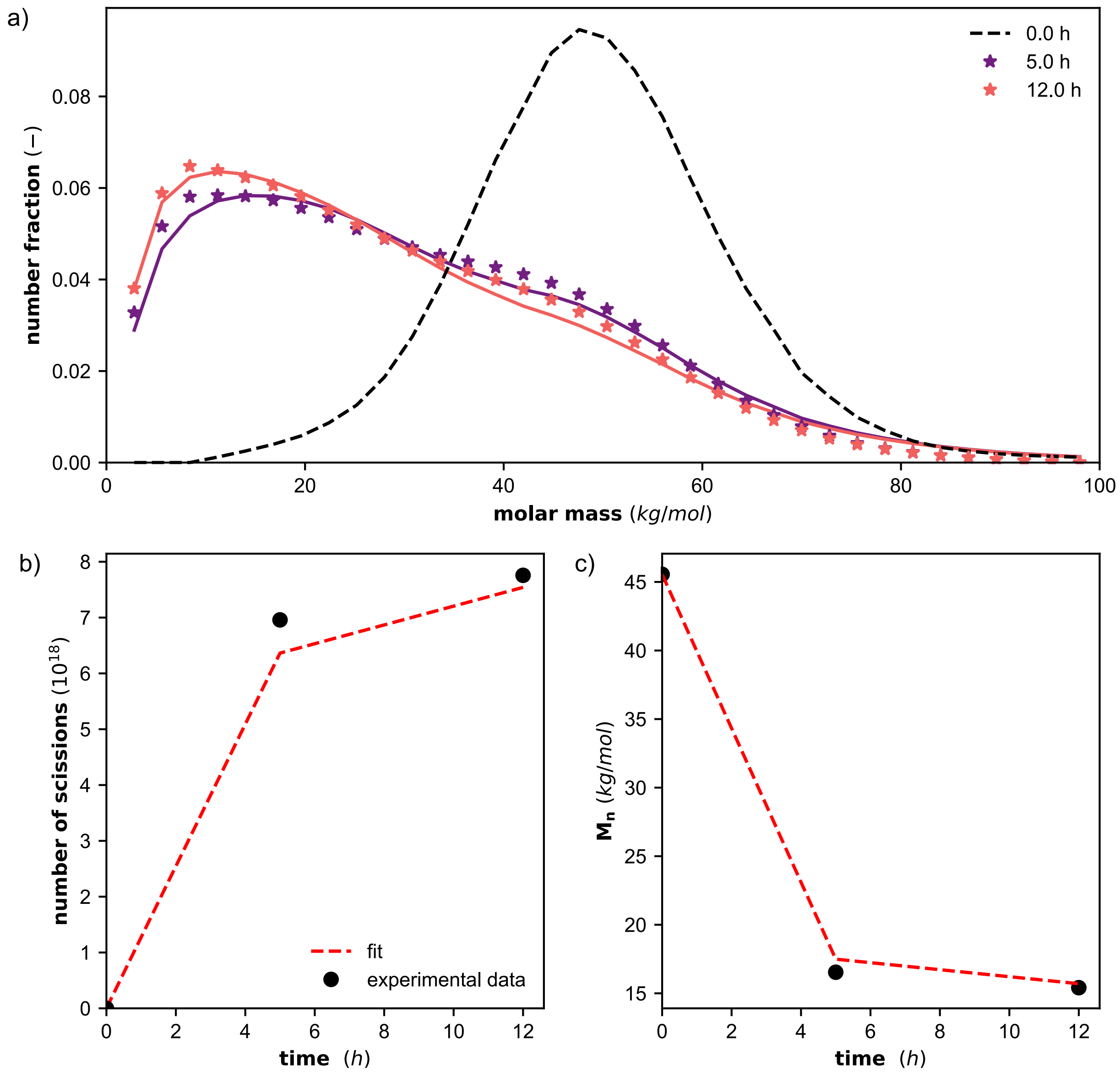

Figure S23. a) Molar mass distributions (solid lines) obtained from HT-SEC before and after milling of 300 mg of PE_{46} for 5, 12 h at 30 Hz at RT under air in a 25 ml steel container, using 5 ZrO_2 grinding spheres (10 mm) together with fits (star symbols) obtained with the model using $N = 30$. $r = 0.18$ $s = 0.00$ are obtained. b) Number of scissions determined by Eq. 1 and c) number averaged molar mass over milling time together with values obtained from the fits.

Milling PE_{103}

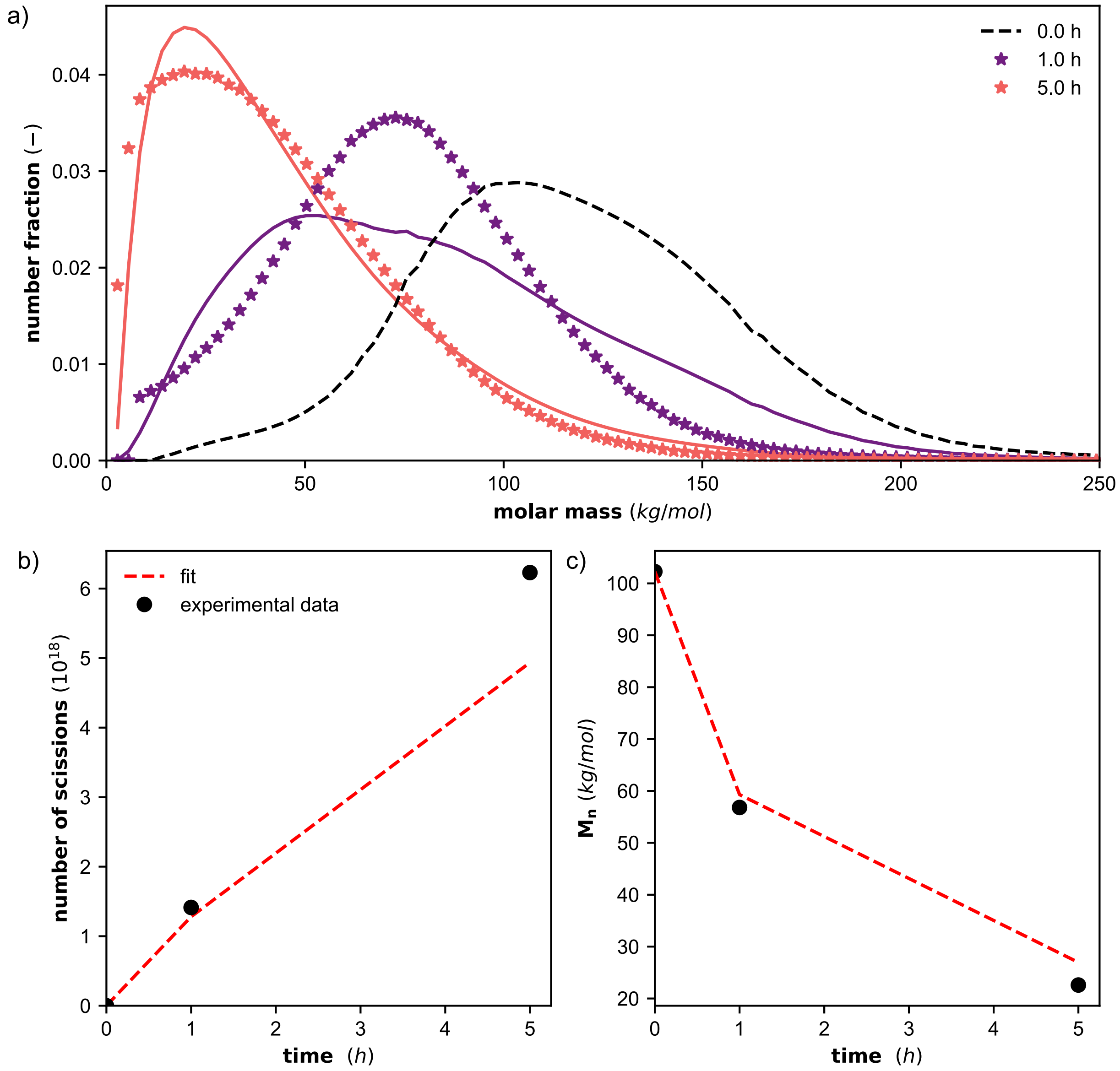

Figure S24. a) Molar mass distributions (solid lines) obtained from HT-SEC before and after milling of 300 mg of PE_{103} for 1 and 5 h at 30 Hz at RT under air in a 25 ml steel container, using 5 ZrO_2 grinding spheres (10 mm) together with fits (star symbols) obtained with the model using $N = 30$. $r = 0.10$ $s = 0.25$ are obtained. b) Number of scissions determined by Eq. 1 and c) number averaged molar mass over milling time together with values obtained from the fits.

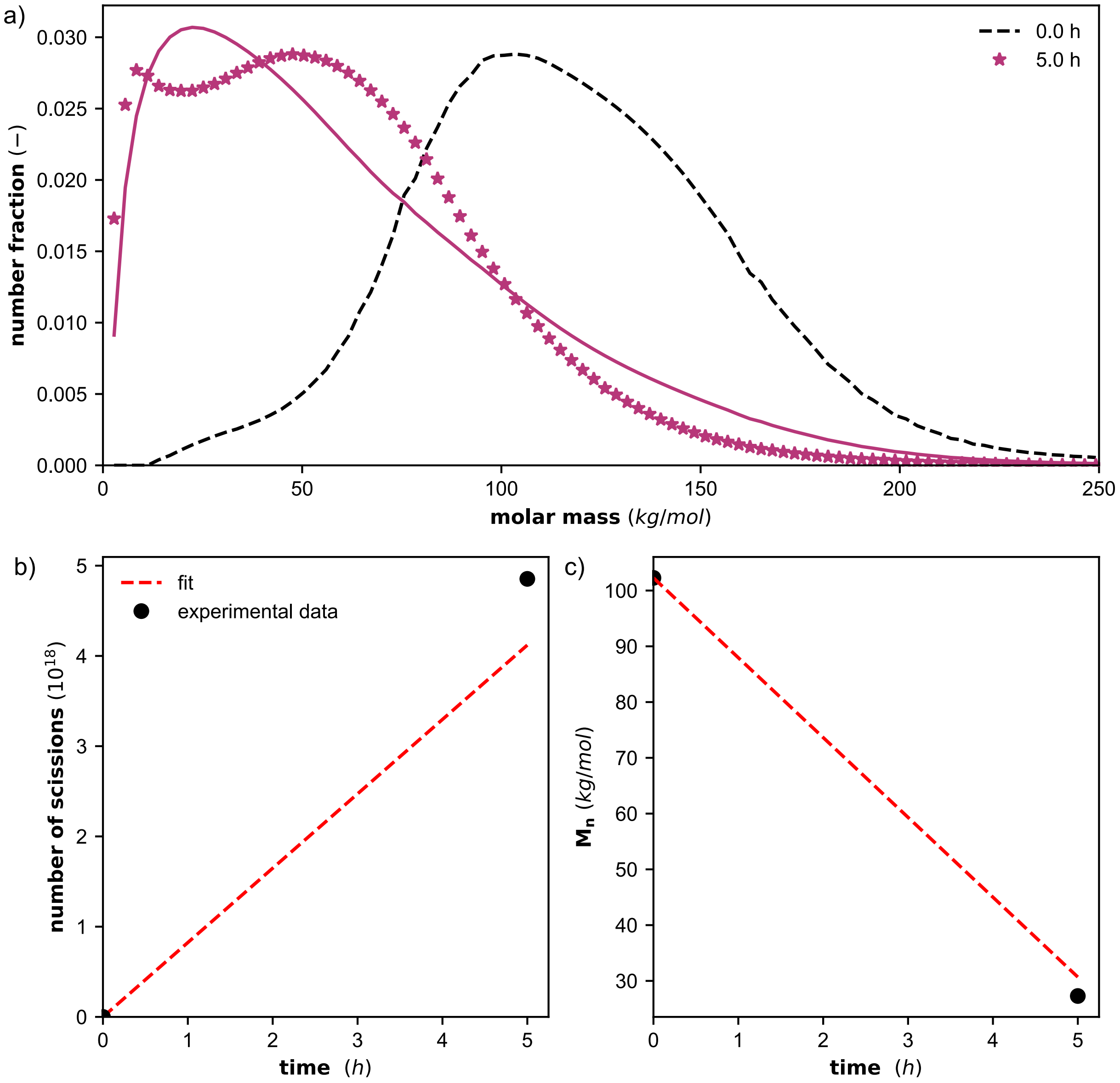

Figure S25. a) Molar mass distributions (solid lines) obtained from HT-SEC before and after milling of 300 mg of PE_{103} for 5 h at 30 Hz at RT under N_2 in a 25 ml steel container, using 5 ZrO_2 grinding spheres (10 mm) together with fits (star symbols) obtained with the model using $N = 30$. $r = 0.15$ $s = 0.00$ are obtained. b) Number of scissions determined by Eq. 1 and c) number averaged molar mass over milling time together with values obtained from the fits.

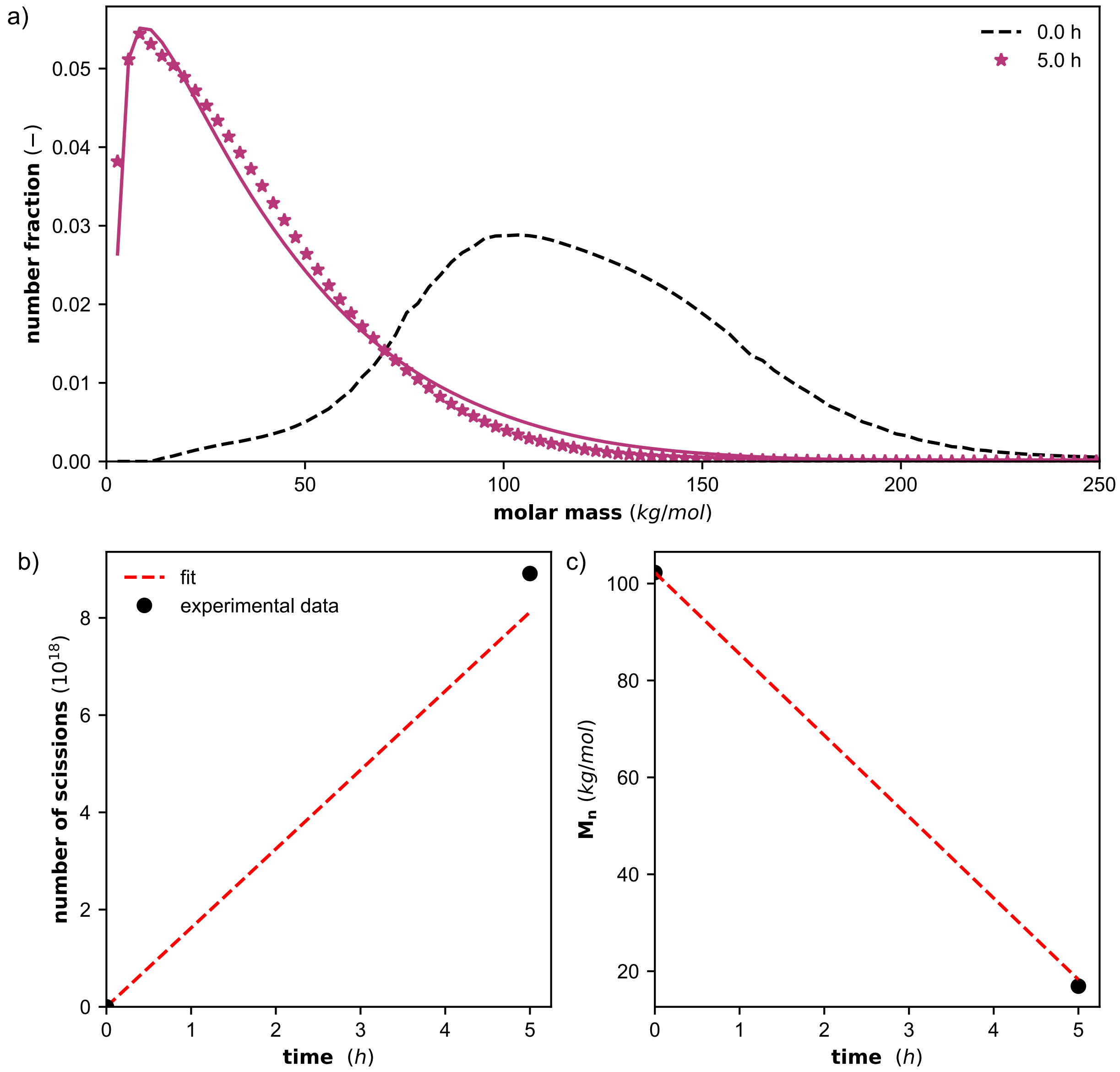

Figure S26. a) Molar mass distributions (solid lines) obtained from HT-SEC before and after milling of 300 mg of PE_{103} for 5 h at 30 Hz at RT under air in a 25 ml steel container, using 5 steel grinding spheres (10 mm) together with fits (star symbols) obtained with the model using $N = 30$. $r = 0.11$ $s = 0.00$ are obtained. b) Number of scissions determined by Eq. 1 and c) number averaged molar mass over milling time together with values obtained from the fits.

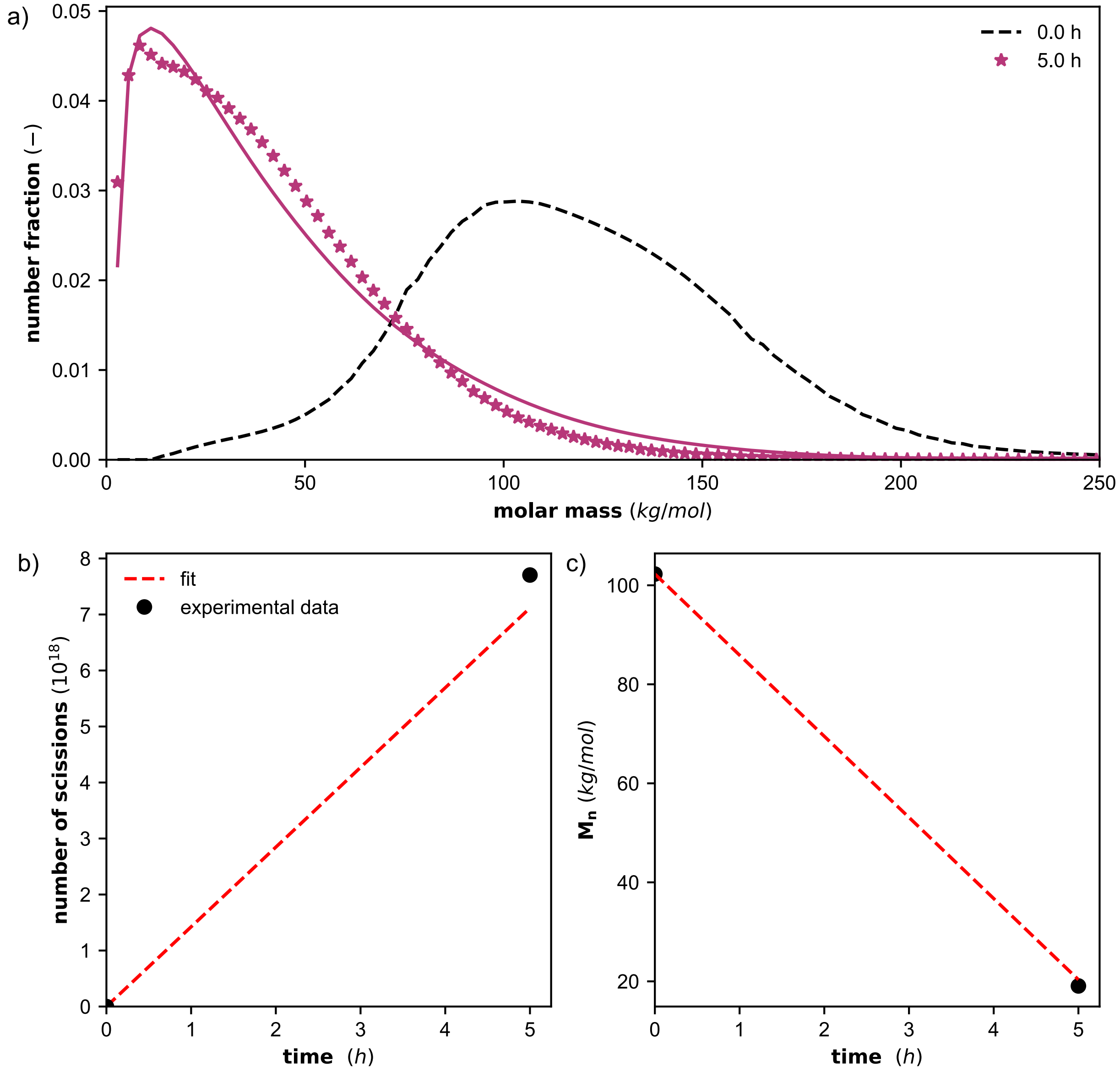

Figure S27. a) Molar mass distributions (solid lines) obtained from HT-SEC before and after milling of 300 mg of PE_{103} for 5 h at 30 Hz at RT under N_2 in a 25 ml steel container, using 5 steel grinding spheres (10 mm) together with fits (star symbols) obtained with the model using $N = 30$. $r = 0.12$ $s = 0.00$ are obtained. b) Number of scissions determined by Eq. 1 and c) number averaged molar mass over milling time together with values obtained from the fits.

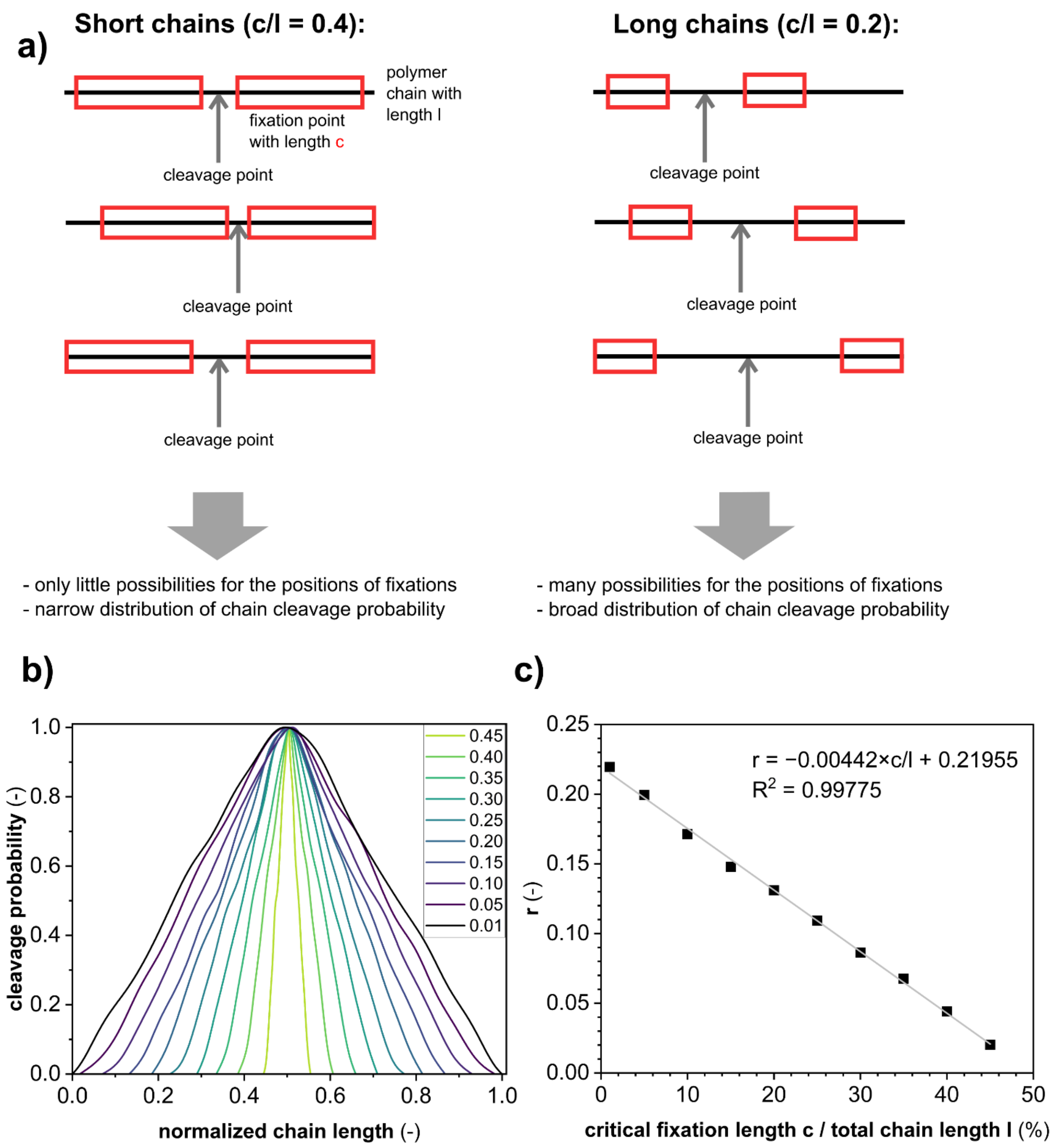

Figure S28. Possible explanation for a correlation of r value and length of the polymer chain l by a simple statistical model assuming a critical minimum fixation chain length c required on both sides of the cleavage point. a) Exemplary illustrations of the possible positions of two fixation blocks of length c on short chains ($c/l = 0.4$) and long chains ($c/l = 0.2$). b) Cleavage probability over normalized chain length for different ratios of c/l. c) Linear correlation of r and c/l. The former was calculated from the widths of the cleavage probability distributions in b).

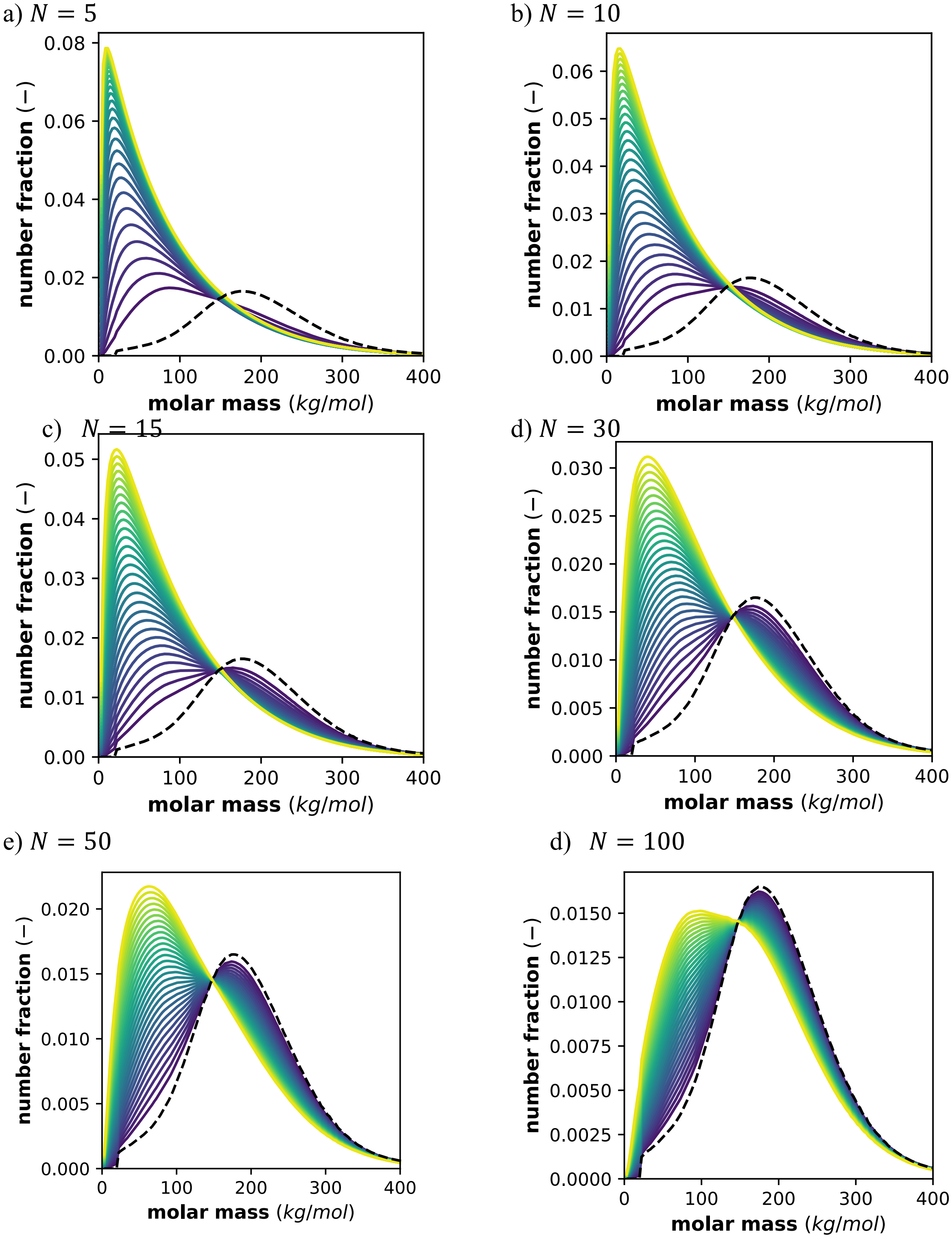
a) $N = 5$
b) $N = 10$
c) $N = 15$
d) $N = 30$
e) $N = 50$
d) $N = 100$
number fraction (−)
molar mass (kg/mol)
0
100
200
300
400

Figure S29. Simulated molar mass distributions over 30 simulation steps with varying values of N. The simulation was performed using $r = 0.14$, $s = 0.18$ and PE_{160} as starting polymer. a) $N = 5$ b) $N = 10$ c) $N = 15$ d) $N = 30$ e) $N = 50$ f) $N = 100$.

S6. Disentangled and annealed polymer and crystallinity

We milled an UHMWPE synthesized in a highly diluted aqueous emulsion polymerization. From this polymerization single chain PE (PE_{single}) nanocrystals are obtained which cannot entangle during growth because of the compartmentalized character of the reaction and a fast crystallization compared to polymerization rate.[14] This polymer, denoted as PE_{single}, has a degree of crystallinity of 71.7 % and an M_n of 1,566 kg/mol. The polymer has a very high melting point of 145.3 ˚C upon first heating at 10 ˚C/min due to the absence of entanglements (**Figure S30a**).[28] After the first melting, entanglements are created which leads to a decreased melting temperature of 136.8 ˚C upon second heating (**Figure S30b**). We subjected PE_{single} to an annealing step at 135 ˚C for 3 h to obtain PE_{anneal}, after which the melting point upon first heating decreased to 133.5 ˚C, demonstrating the presence of entanglements. In addition, the degree of crystallinity decreased to 53.4 %. (**Figure S30a**).

As is expected for a disentangled polymer, with a slower heating rate of 1 ˚C/min, the melting temperature of PE_{single} decreases to 136.8 ˚C upon first heating (**Figure S30c**).[28] With the slower rate, the chains have more time to unfold. In contrast, an entangled polymer would not show such a decrease in melting temperature, because the entanglements hinder unfolding. However, unexpectedly, the annealed sample, denoted as PE_{anneal} melts at an even lower temperature than PE_{single} with 1 ˚C/min heating rate. This could be due to a decrease in crystal size upon annealing for which the decreased crystallinity could be indicative. Interestingly, the melting temperature after milling increases for both PE_{anneal} and PE_{single}.

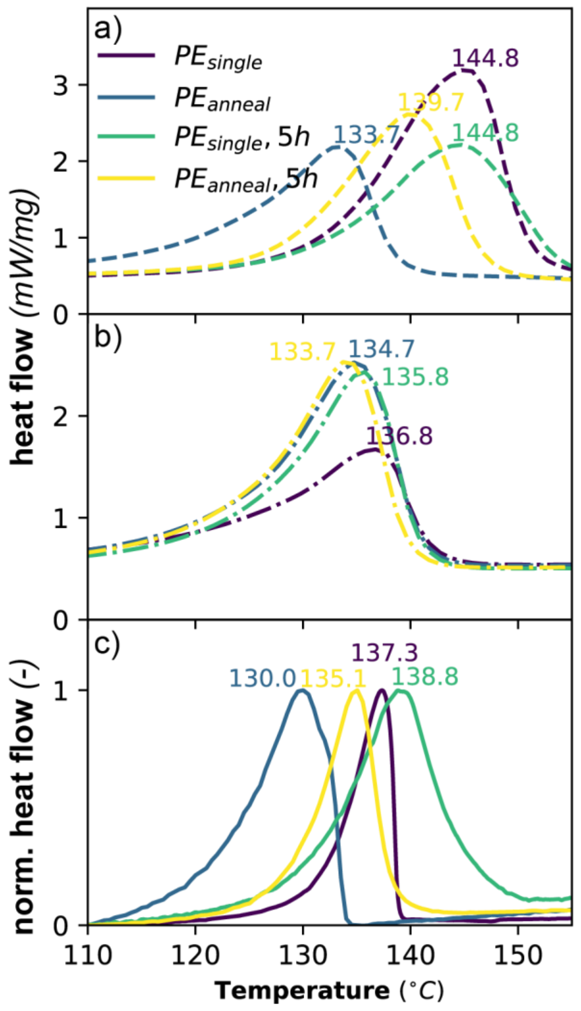

Figure S30. DSC curves of untreated, annealed UHMWPE and both samples after milling at 30 Hz, at RT under N_2 for 5 h using ZrO_2 spheres: a) first heat at 10 ˚C/min, b) second heat at 10 ˚C/min, c) first heat at 1 ˚C /min.

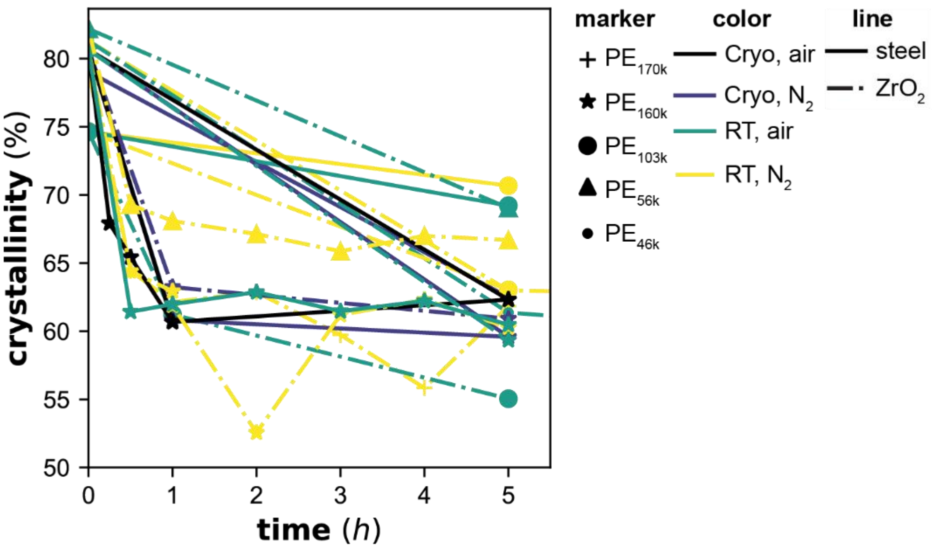

Figure S31. Degree of crystallinity before and after milling under various conditions.
